# Alternative splicing of *ceramide synthase 2* alters levels of specific ceramides and modulates cancer cell proliferation and migration in Luminal B breast cancer subtype

**DOI:** 10.1038/s41419-021-03436-x

**Published:** 2021-02-10

**Authors:** Trishna Pani, Kajal Rajput, Animesh Kar, Harsh Sharma, Rituparna Basak, Nihal Medatwal, Sandhini Saha, Gagan Dev, Sharwan Kumar, Siddhi Gupta, Arnab Mukhopadhyay, Dipankar Malakar, Tushar Kanti Maiti, Aneeshkumar G. Arimbasseri, S. V. S. Deo, Ravi Datta Sharma, Avinash Bajaj, Ujjaini Dasgupta

**Affiliations:** 1 Amity Institute of Integrative Sciences and Health, Amity University Haryana, Panchgaon, Manesar, Gurgaon, 122413 Haryana India; 2 Regional Centre for Biotechnology, NCR Biotech Science Cluster, 3rd Milestone Faridabad-Gurgaon Expressway, Faridabad, 121001 Haryana India; 3 Manipal Academy of Higher Education, Manipal, 576104 Karnataka India; 4 National Institute of Immunology, Aruna Asaf Ali Marg, New Delhi, 110067 India; 5 SCIEX, 121 Udyog Vihar, Phase IV, Gurgaon, 122015 Haryana India; 6 Department of Surgical Oncology, BRA-Institute Rotary Cancer Hospital, All India Institute of Medical Sciences, New Delhi, 110029 India; 7 Present Address: Kusuma School of Biological Sciences, Indian Institute of Technology, New Delhi, 110016 India

**Keywords:** Breast cancer, Cancer metabolism

## Abstract

Global dysregulation of RNA splicing and imbalanced sphingolipid metabolism has emerged as promoters of cancer cell transformation. Here, we present specific signature of alternative splicing (AS) events of sphingolipid genes for each breast cancer subtype from the TCGA-BRCA dataset. We show that *ceramide synthase 2* (*CERS2*) undergoes a unique cassette exon event specifically in Luminal B subtype tumors. We validated this exon 8 skipping event in Luminal B cancer cells compared to normal epithelial cells, and in patient-derived tumor tissues compared to matched normal tissues. Differential AS-based survival analysis shows that this AS event of *CERS2* is a poor prognostic factor for Luminal B patients. As Exon 8 corresponds to catalytic Lag1p domain, overexpression of AS transcript of *CERS2* in Luminal B cancer cells leads to a reduction in the level of very-long-chain ceramides compared to overexpression of protein-coding (PC) transcript of *CERS2*. We further demonstrate that this AS event-mediated decrease of very-long-chain ceramides leads to enhanced cancer cell proliferation and migration. Therefore, our results show subtype-specific AS of sphingolipid genes as a regulatory mechanism that deregulates sphingolipids like ceramides in breast tumors, and can be explored further as a suitable therapeutic target.

## Introduction

Sphingolipids help in maintaining the structural integrity of cell membranes, and also aid in numerous signaling processes in response to different stimuli^1–3^. Dysregulation of the sphingolipid pathway is one of the important contributing factors for breast cancer pathogenesis as it is implicated in various aspects of cancer initiation, progression, invasion, metastasis, and drug resistance^4,5^. Any alteration in the enzymes regulating the expression of sphingolipids play a vital role in cancer cell survival and apoptosis^6^. Therefore, dynamic metabolic interconversions among sphingolipids, attuning the cellular signaling mechanisms in cancer cells, make them a desirable yet challenging target for cancer therapy^7,8^.

Ceramides are key precursors of complex sphingolipids that are synthesized via de novo as well as through salvage pathway. De novo pathway involves conjugation of specific fatty acyl chains to sphinganine using ceramide synthases, whereas salvage pathway involves degradation of sphingomyelins and complex sphingolipids to ceramides^9^. Human breast cancer tissues usually have high expression of ceramide species as compared to normal tissue samples^10^. There is an increased expression of C16:0, C24:0, and C24:1 ceramides in malignant tumors as compared to benign and normal tissues, where C16:0 ceramides were associated with positive lymph node status^11^. Estrogen receptor (ER)-positive breast tumors showed high expression of C18:0 and C20:0 ceramides as compared to ER-negative tumor tissues^11^.

Mammalian cells express six ceramide synthase isoforms (*CERS*1-6) that show similar catalytic mechanism and intracellular localization in the endoplasmic reticulum but exhibit distinct fatty acyl-CoA specificity^12^. As CERSs are key enzymes of sphingolipid metabolism, many genetic, transcriptional, post-transcriptional, and post-translational regulatory mechanisms regulating *CERSs* have been identified in various pathophysiological processes^13^. *CERSs* including *CERS2*, *CERS4*, and *CERS6* are overexpressed in malignant breast tissues^14,15^ Human bladder carcinoma patients with loss of *CERS2* mRNA expression have poor prognosis, and is associated with tumor progression and invasion^16^. *CERS2* mRNA levels are also found to be low among high-grade meningioma patients who suffer from tumor recurrence more frequently, and have increased risk of death^17^.

*CERS2* is the only ceramide synthase that codes for very-long-chain ceramides. As altered expression of *CERS2* is associated to different types of cancer^18^, deciphering the mechanisms responsible for dysregulation of *CERS2* gene expression is critical for understanding its role in cancer progression. Exon 1 of *CERS2* has been shown to regulate the *CERS2* transcription as it allows the binding of Kruppel-like factor 6 (KLF6) and zinc finger transcription factor, Sp1 (ref. ^19^). This transcription factor-mediated upregulation of *CERS2* is effective in suppression of metastasis of prostate cancer cells. Among different post-transcriptional regulatory mechanisms, miR-133a, miR-221, and miR-222 have been identified to bind with 3′-UTR regions of *CERS2*, and regulate its expression^20,21^. A truncated CERS2, tumor metastasis suppressor gene-1 (TMSG1), lacking the N-terminal 150 amino acid residues showed very low expression in several metastatic cancer cell lines and tissues compared to the non-metastatic ones^22^. Although other *CERS2* splice variants have also been reported, *CERS2* alternative transcript lacking exon 8 has not been studied in detail.

Alternative splicing (AS) has emerged as a crucial post-transcriptional regulatory mechanism in many disease conditions, especially cancer^23^. Reorganization and differential expression of specific transcript isoforms are beneficial for cancer pathogenesis, making them a valuable target for cancer therapy^24^. AS also modifies the balance between pro-survival and pro-apoptotic variants of many proteins like *p53*, *caspase 2*, *FAS*, *BCL2L1*, and *caspase 9 (*ref. ^25,26^). Recently, our group has shown that a combination chemotherapy induces the AS in glucocerebrosidase β (*Gba1*), and increases Gba1 expression in murine tumor tissues. This enhanced Gba1 expression catalyzes the degradation of glucosylceramides to ceramides responsible for tumor regression, and lowers the glucosylceramide levels critical for drug resistance^27^.

Recent studies have shown that subtype-specific qualitative and quantitative expression of different transcript isoforms via AS are integral to the molecular portrait of each subtype in breast cancer patients^28,29^. Differential expression of dysregulated transcript isoforms along with prominent isoform switching patterns have allowed stratification of subtypes in breast cancer patients^30^. We hypothesize that different breast cancer subtypes (Luminal A, Luminal B, HER2^+^, and Basal) may possess unique RNA splicing signatures for genes of the sphingolipid pathway, and post-transcriptional regulation (alternative splicing) of these genes, especially ceramide synthases, may contribute to breast cancer development.

Therefore, we aimed to identify the AS events of sphingolipid genes in different breast cancer subtypes from TCGA-BRCA dataset^31^. We show that *ceramide synthase 2* (*CERS2*) undergoes a unique AS event where Exon 8 is skipped in Luminal B subtype patients, and validate the differential expression of *CERS2* isoforms in Luminal B representative cancer cells and tumor tissues. We further show that this AS event in *CERS2* significantly affects survival in Luminal B patients of TCGA-BRCA cohort, and is a poor prognosis factor. Loss of Exon 8 contributes to the lack of catalytic activity and substrate specificity of CERS2 for very-long-chain ceramides. Finally, we demonstrate that loss of Exon 8 reduces the levels of very-long-chain ceramides, thereby affecting the cancer cell proliferation and migration.

## Results

### Breast cancer patients exhibit subtype-specific AS events

**Fig. 1:**
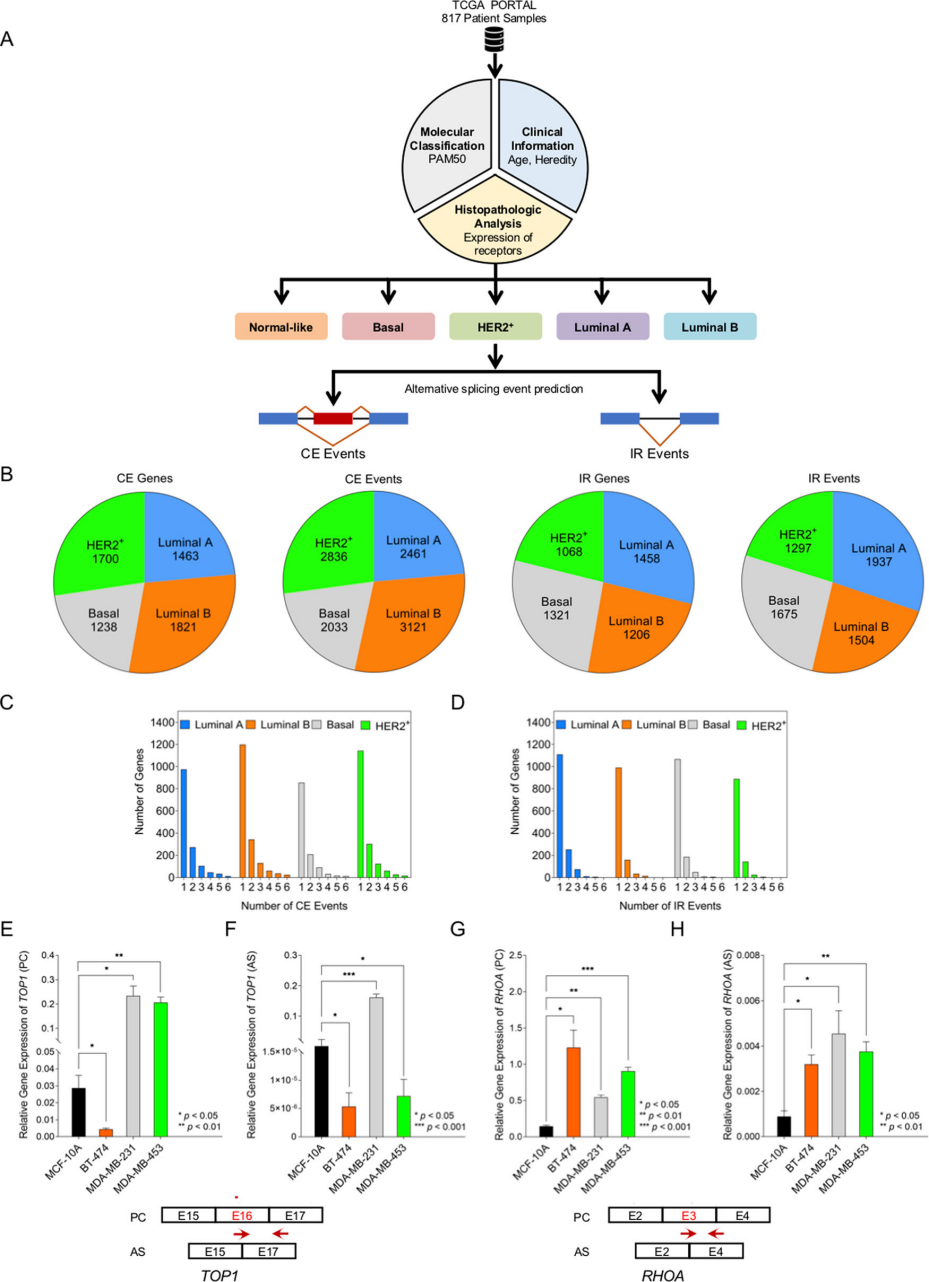
Breast cancer patients exhibit subtype-specific alternative splicing (AS) events. **A** Schematic representation of the strategy for identifying alternatively spliced (AS) events in breast cancer subtypes (TCGA-BRCA dataset). **B** Number of AS events caused by differential usage of cassette exons (CE) or by intron retention (IR) for Luminal A, Luminal B, HER2^+^, and Basal breast cancer subtypes, and total number of genes that they correspond to. Statistical test Irwin–Hall *p*-value summarization was used on individual splicing events. Details of samples for each subtype are in Data set 1 in supplementary information. **C**, **D** Distribution of number of genes undergoing different number of CE (**C**) and IR events (**D**) predicted for Luminal A, Luminal B, HER2^+^, and Basal subtypes. **E**–**H** Quantitative real-time-PCR (qRT-PCR) validation in MCF-10A (untransformed mammary epithelial-derived), and representative subtype-specific cancer cell lines like BT-474 (Luminal B), MDA-MB-231 (Basal), and MDA-MB-453 (HER2^+^) for CE event in *TOP1* (**E**, **F**) predicted for Luminal B and HER2^+^ subtypes; and for CE event in *RHOA* (**G**, **H**) predicted for Basal subtype. Normalized transcript level for protein-coding (PC) and alternatively spliced (AS) isoforms of *TOP1* and *RHOA* gene for each cell line (mean ± SD, *n* = 4) show differential expression of the isoforms. A pictorial representation of exon/intron positions and primer sites are also shown. Data in (**E**–**H**) were analyzed by ANOVA.

RNA sequencing (RNA-Seq) data for TCGA-BRCA controlled dataset was downloaded from Genomics Data Commons (GDC portal, NIH) for Breast Invasive Carcinoma Project^31^. The data for 817 cases of Invasive Ductal Carcinoma (IDC) was classified into five different subtypes (Luminal A, Luminal B, HER2^+^, Basal, and Normal-like) based on their hormonal character and PAM50 gene expression (Fig. 1A, Data set 1). We analyzed the RNA-Seq data to identify transcriptome-wide splicing events in different breast cancer subtypes compared to Normal-like using a previously described bioinformatic pipeline^32^. We used ‘Normal-like’ as the reference based on the understanding that the “Normal-like” tumors probably had only a few tumor cells and a large number of normal breast epithelium^33^, and therefore, they have a profile measuring nearest to the normal breast epithelium. Our analysis specifically focussed on two AS events, cassette exon (CE) events where an exon is spliced-in or spliced-out, and intron retention (IR) events where an intron is retained in an isoform under a certain condition (Fig. 1A).

We recorded higher incidence of CE events in all four subtypes as compared to IR events. There are 2461 CE events in 1463 genes, and 1937 IR events in 1458 genes in Luminal A subtype. There are 3121 CE events in 1821 genes, and 1504 IR events in 1206 genes in Luminal B subtype (Fig. 1B, Data set 2, 3). In contrast, there are 2033 CE events in 1238 genes and 1675 IR events in 1321 genes in Basal subtype, and 2836 CE events in 1700 genes and 1297 IR events in 1068 genes in HER2^+^ subtype (Fig. 1B, Data set 2, 3). The number of CE and IR events per gene varied in different subtypes with maximum number of genes undergoing only one AS event, and number of genes progressively decreases with increase in frequency of AS events (Fig. 1C, D).

AS in cancer-related genes can alter the desired protein expression and modulate the signal transduction pathways, thereby leading to transformation of cancer cells^34^. Gene enrichment analysis using DAVID showed that subtype-specific genes predicted to undergo AS are enriched for metabolic pathways, cell cycle arrest, transport and trafficking, DNA repair, protein homeostasis, and mRNA transport and splicing (Supplementary Figures 1–4, Data sets 4–7). GO terms for the predicted AS genes showed biological processes such as mitosis, cell adhesion, GTPase and transcription factor-mediated signaling pathways, translation, and transport (Supplementary Figures 1–4, Data sets 4–7). Protein domain enrichment analysis of AS genes showed inclusion of WD 40 repeat domain, Pleckstrin homology domain, Ubiquitin-interacting motif, Armadillo-type fold, and Zinc finger motifs that are involved in protein targeting/trafficking, cell adhesion, and protein catabolism and signaling suggesting that these domains may perform well-assigned functions in cancer cell growth and signaling (Supplementary Figures 1–4, Data sets 4–7).

We validated two predicted splicing events from TCGA data sets in representative breast cancer subtype cell lines like BT-474 (Luminal B), MDA-MB-231 (TNBC/Basal), and MDA-MB-453 (HER2^+^). We used normal epithelial mammary MCF-10A cells as control. A CE event in the Exon 16 of *Topoisomerase1* (*TOP1*) was identified for Luminal B and HER2^+^ subtypes (*p* < 0.0001) where Exon 16 is retained. *TOP1* plays an essential role in alleviating topological stress arising during DNA replication and transcription^35^. We used event-specific primer pairs for the predicted CE event, and found that levels of protein-coding (PC) and AS transcripts varied in different cell lines (Fig. 1E, F). The level of AS transcript is significantly lower in BT-474 (*p* < 0.05) and MDA-MB-453 (*p* < 0.05) cells as compared to MCF-10A cells, but higher in MDA-MB-231 cells (*p* < 0.001) (Fig. 1F, Data set 2, Supplementary Table 1).

We also observed a CE event targeting Exon 3 of a Ras homolog family member A (*RHOA*) in basal subtype (*p* < 0.0003) where Exon 3 is skipped. *RHOA* is a small GTPase protein known to regulate malignant transformation and motility of cancer cells^36^. Using event-specific primer pairs, we observed differential expression of PC and AS isoforms in all cell lines (Fig. 1G, H, Data set 2, Supplementary Table 1). The level of AS transcripts that include the predicted event (Exon 3 exclusion) is significantly higher in all subtype-specific cell lines with MDA-MB-231 cells showing the most abundant AS1 transcripts (*p* < 0.05), followed by BT-474 cells (*p* < 0.05), and MDA-MB-453 cells (*p* < 0.01) as compared to MCF-10A cells (Fig. 1H).

### AS of sphingolipid pathway genes provide subtype-specific signature

**Fig. 2:**
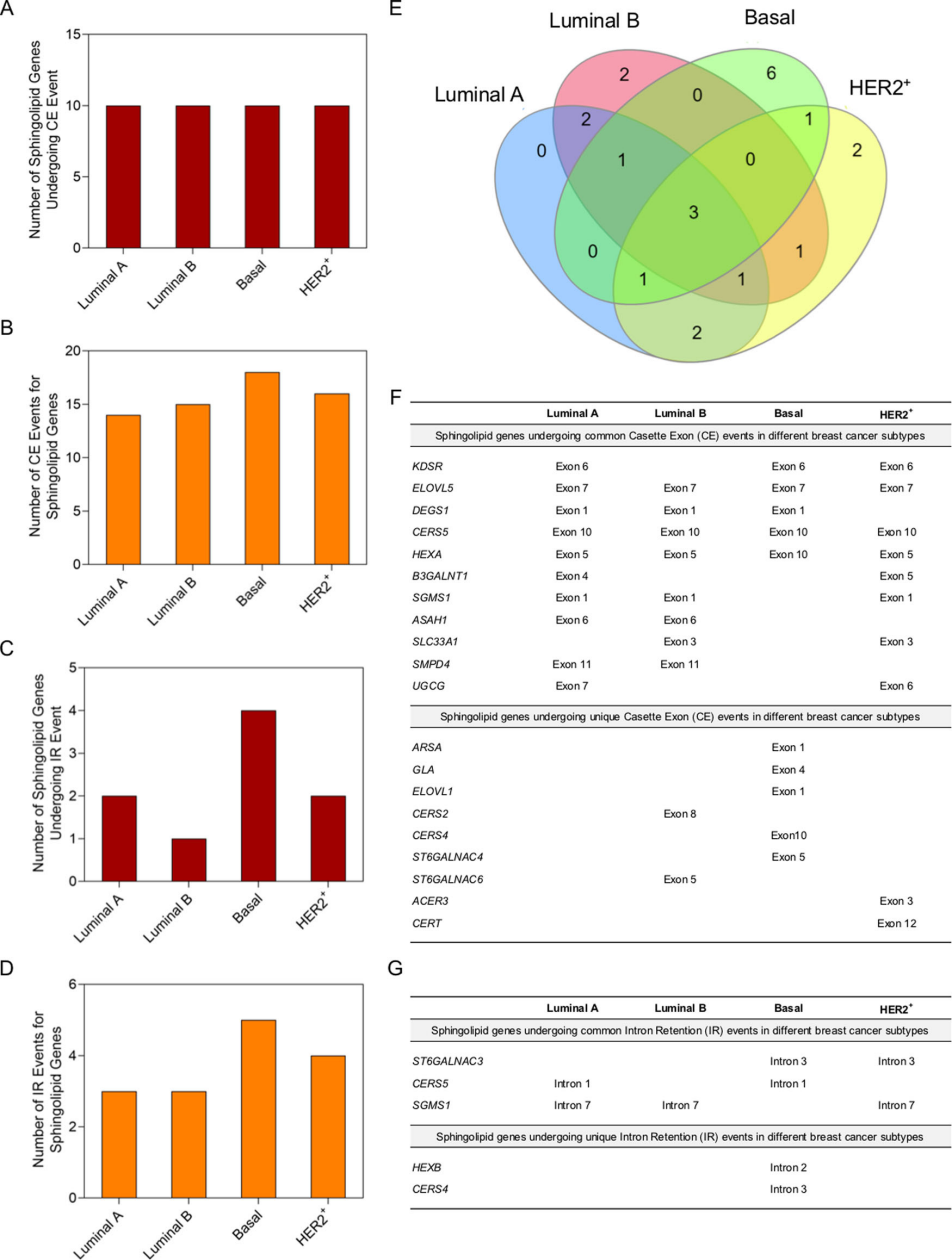
Alternative Splicing in sphingolipid genes generate a breast cancer subtype-specific signature. **A**–**D** Number of AS events in sphingolipid-metabolizing genes in breast cancer subtypes (TCGA-BRCA dataset) compared to normal-like breast tissue samples, as shown by differential usage of cassette exons (CE) (**A**, **B**) or by intron retention (IR) (**C**, **D**) events. Total number of Cassette Exon (CE) (**B**) and Intron Retention (IR) (**D**) events are shown for Luminal A, Luminal B, HER2^+^, and Basal subtypes, and total number of genes that they correspond to (**A**, **C**). Statistical test Irwin–Hall *p*-value summarization was used on individual splicing events. **E** Venn diagram showing the number of common and unique AS events in sphingolipid genes in different breast cancer subtypes (TCGA-BRCA dataset). **F**, **G** List of all CE (**F**) and IR events (**G**) in different sphingolipid-metabolizing genes in different breast cancer subtypes suggesting a subtype-specific AS signature.

Analysis of subtype-specific CE and IR events specifically for genes of sphingolipid pathway revealed 18 CE events in 10 genes and 5 IR events in 4 genes in Basal subtype, and 14 CE events in 10 genes and 3 IR events in 2 genes for Luminal A subtype (Fig. 2A–D). We also observed 15 CE events in 10 genes and 3 IR events in 1 gene in Luminal B subtype, and 16 CE events in 10 genes and 4 IR events in 2 genes in HER2^+^ subtype (Fig. 2A–D). Maximum number of sphingolipid genes that undergo AS were predicted to have one event while few selected genes showed two or three events (Data set 8, 9). Sphingolipid genes undergoing AS showed some common signatures for all the subtypes as well as a unique signature for each subtype (Fig. 2E, Data set 8, 9). For example, common AS targets among subtypes include CE events in *Fatty acid elongase 5* (*ELOVL5*), *CERS5*, and *Hexosaminidase subunit alpha* (*HEXA*) (Fig. 2F). In contrast, CE event in *CERS4* is specific for Basal subtype (Fig. 2F), whereas CE event in *CERS2* is unique for Luminal B subtype (Fig. 2F). Among different IR events, IR event in *CERS5* is common between Luminal A and Basal subtypes, and there is an IR event in *CERS4* that is unique for Basal subtype (Fig. 2G). As ceramide is the key precursor of sphingolipid pathway, AS events in *CERS* genes may regulate the subtype-specific synthesis of ceramides and other sphingolipids in breast cancer patients. Subtype-specific AS events in different *CERS* genes such as *CERS2* in Luminal B subtype and *CERS4* in Basal subtype suggest a strong subtype-specific post-transcriptional regulation of *CERS* genes that may play a role in breast cancer pathogenesis (Fig. 2F, G; Data set 8, 9).

### *CERS2* is alternatively spliced in Luminal B subtype

**Fig. 3:**
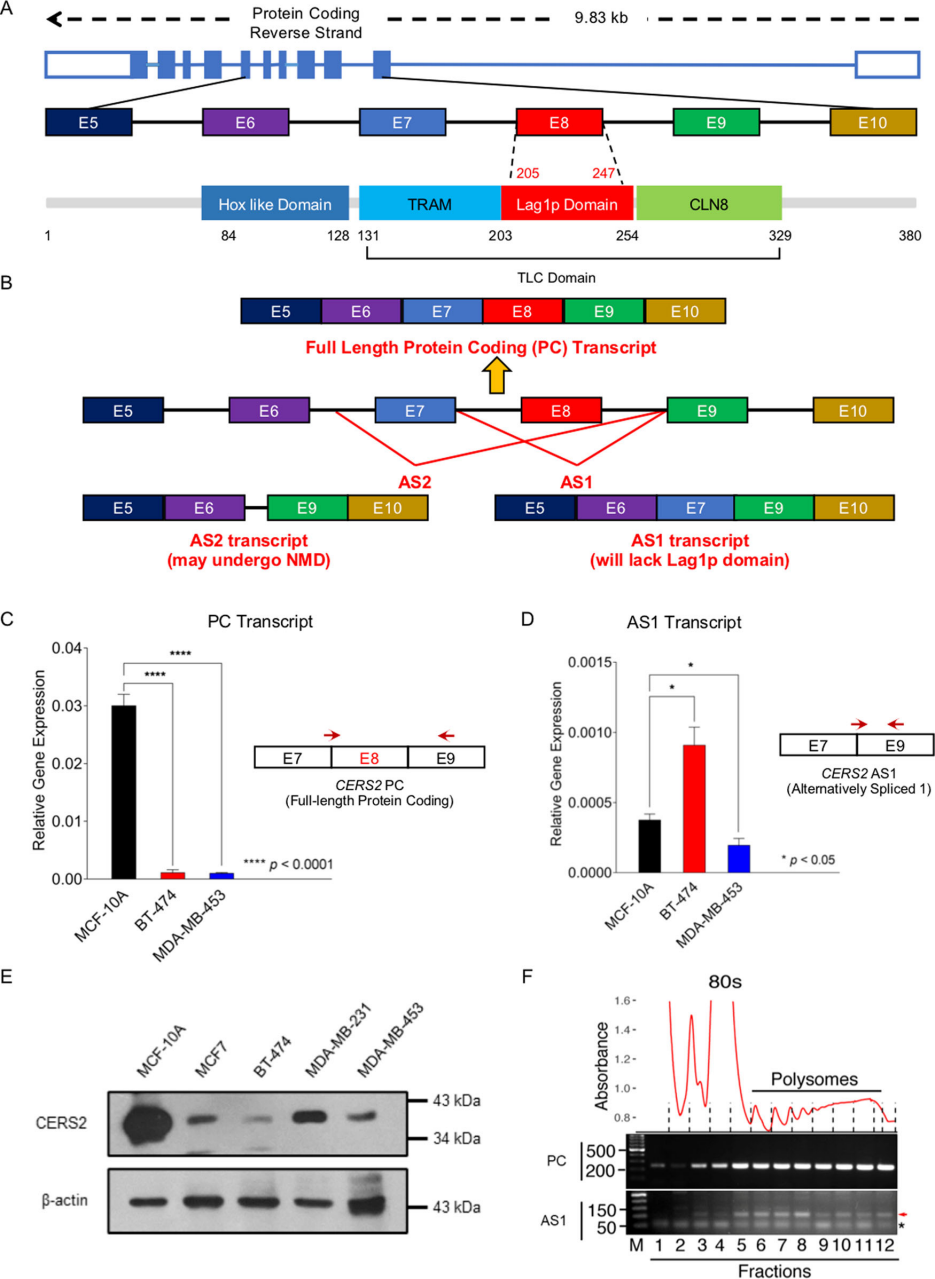
Exon 8 skipping in *ceramide synthase 2* (*CERS2*) is a specific event for Luminal B subtype. **A** Pictorial representation of *CERS2* gene and corresponding protein showing the distinct Hox-like domain, Tram-Lag-CLN8 (TLC) domain, and Lag1p motif. **B** Two CE events predicted for *CERS2* where AS1 generates a transcript in which exon 8 (205–247 amino acids) is skipped, and AS2 event generates a transcript with 33 bp of intron 6–7 retained and loss of exon 7 and 8 (174–247 amino acids). **C**, **D** Isoform-specific qRT-PCR (mean ± SD, *n* = 6) for *CERS2* full-length protein-coding (PC) transcript (**C**) and alternatively spliced AS1 transcript lacking Exon 8 (**D**) in MCF-10A, BT-474 (Luminal B), and MDA-MB-453 (HER2^+^) cells confirm differential expression of the isoforms in BT-474 cells as compared to MCF-10A and MDA-MB-453 cells. A pictorial representation of exon/intron position and primer sites are also shown. **E** Immunoblot of CERS2 from MCF-10A and representative subtype-specific breast cancer cells show a single band corresponding to full-length protein-coding (PC) CERS2 protein. **F** Polysome analysis of BT-474 cells shows A260 tracing of fractions in upper panel. Bottom panel shows qRT-PCR analysis of fractions as listed at the bottom of the gel image. Panel PC shows full-length protein-coding (PC) mRNA specific product, and panel AS shows alternatively spliced mRNA (AS1) specific product. Red arrow indicates correct size for the alternate spliced mRNA in the polysome fractions while the asterisk indicates a non-specific product. Numbers on the left-hand side of the gel indicate the length of marker DNA in base pairs. Data in (**C**, **D**) were analyzed by ANOVA.

Bioinformatic analysis identified an alternatively spliced transcript (AS1 transcript) for *CERS2* (*ENSG0000143418*) in Luminal B subtype tumors that skips Exon 8 (*p* = 0.030) (Fig. 3A, B, Supplementary Figures 5–7, Data set 8). Another transcript predicted for *CERS2* (AS2 transcript) includes a portion of the intron between Exons 6 and 7, and excludes Exons 7 and 8 (*p* < 0.05) (Fig. 3B, Supplementary Figures 8–10). AS2 transcript shows a premature stop codon (Supplementary Figure 10), and is probably subjected to nonsense-mediated decay (NMD) through the NMD pathway^37^. Therefore, we focused on the rest of our study on the full-length PC and the alternatively spliced, AS1, transcript of *CERS2*.

We validated the AS1 event in Luminal B BT-474 cells, and compared it with HER2^+^ MDA-MB-453 and normal MCF-10A cells using specific primer pairs of similar efficiency for PC and AS1 transcripts (Supplementary Table 1). The PC transcript is highly abundant in MCF-10A cells in comparison to BT-474 and MDA-MB-453 cells (Fig. 3C), whereas the AS1 transcript is significantly more in BT-474 cells and not in MDA-MB-453 cells as compared to MCF-10A cells (Fig. 3D). As the abundance of *CERS2* AS1 transcript is very low, it was not possible to use the canonical approaches of using one primer pair to identify both PC and AS1 transcript isoforms. We, therefore, used a strategy based on published methods, that has shown the use of boundary-spanning (exon–exon junction specific) primers to quantify the different isoforms of a transcript that differ in their abundance^38–40^. However, using RNA isolated from cells overexpressing PC and AS1 transcripts, both transcripts can be identified using the same primer pair. We used forward primer from exon 7 and reverse primer from exon 9, thereby giving a 129 bp reduction in band size for AS1 overexpressing BT-474 cells (Supplementary Figure 11).

CERS2 is a transmembrane protein of 380 amino acids with a 199 amino acids long Tram-Lag-CLN8 (TLC) domain (Fig. 3A). Lag1p motif, a conserved stretch of 52 amino acids (203–254), is a part of the 151 residues (151–301 amino acids) span of TLC domain that is predicted to be the minimal region required for acyl chain specificity^41,42^ (Fig. 3A, Supplementary Figure 12). Lag1p motif, with two conserved histidine residues, is also suggested to be crucial for CERS catalytic activity and substrate binding (Supplementary Figure 13)^42^. Structure-function relationships of CERS proteins have found that Lag1p motif confers catalytic activity, and assists in substrate binding to synthesize very-long-chain (C22:0, C24:0, C24:1) ceramides for CERS2^43^. As Exon 8 of CERS2 corresponds to a stretch of 43 amino acids (205–247) of Lag1p motif, AS1 transcript lacking Exon 8 would code for a protein devoid of almost the entire Lag1p domain (Supplementary Figure 12).

Immunoblot of MCF-10A, BT-474, MDA-MB-231, and MDA-MB-453 cell lysates with CERS2 antibody showed a higher expression of CERS2 protein (band closer to 43 kDa) arising from full-length PC transcript (Fig. 3E). Among different breast cancer cell lines, BT-474 cells showed the lowest CERS2 expression arising from full-length PC transcript. Repeated attempts to visualize the protein product from AS1 transcript (5 kDa smaller than PC protein) on an immunoblot were unsuccessful due to low abundance of the AS1 transcript as compared to the PC transcript. However, we could detect both PC and AS1 protein products translated from overexpression of PC and AS1 transcripts in HEK 293T cells (Supplementary Figure 14), thereby validating that AS1 transcript gets translated. If AS1 transcript indeed gets translated in BT-474 cells, corresponding mRNAs will be associated with polysomes. Therefore, we analyzed the distribution of full-length PC and AS1 mRNAs in the polysome fractions of BT-474 cells. As expected, full-length PC mRNA is distributed throughout the polysomes. Interestingly, we observed that AS1 mRNA is also enriched specifically in the polysome fractions (fractions 5–12) compared to free RNA or monosome fractions (fractions 1–4) (Fig. 3F).

**Fig. 4:**
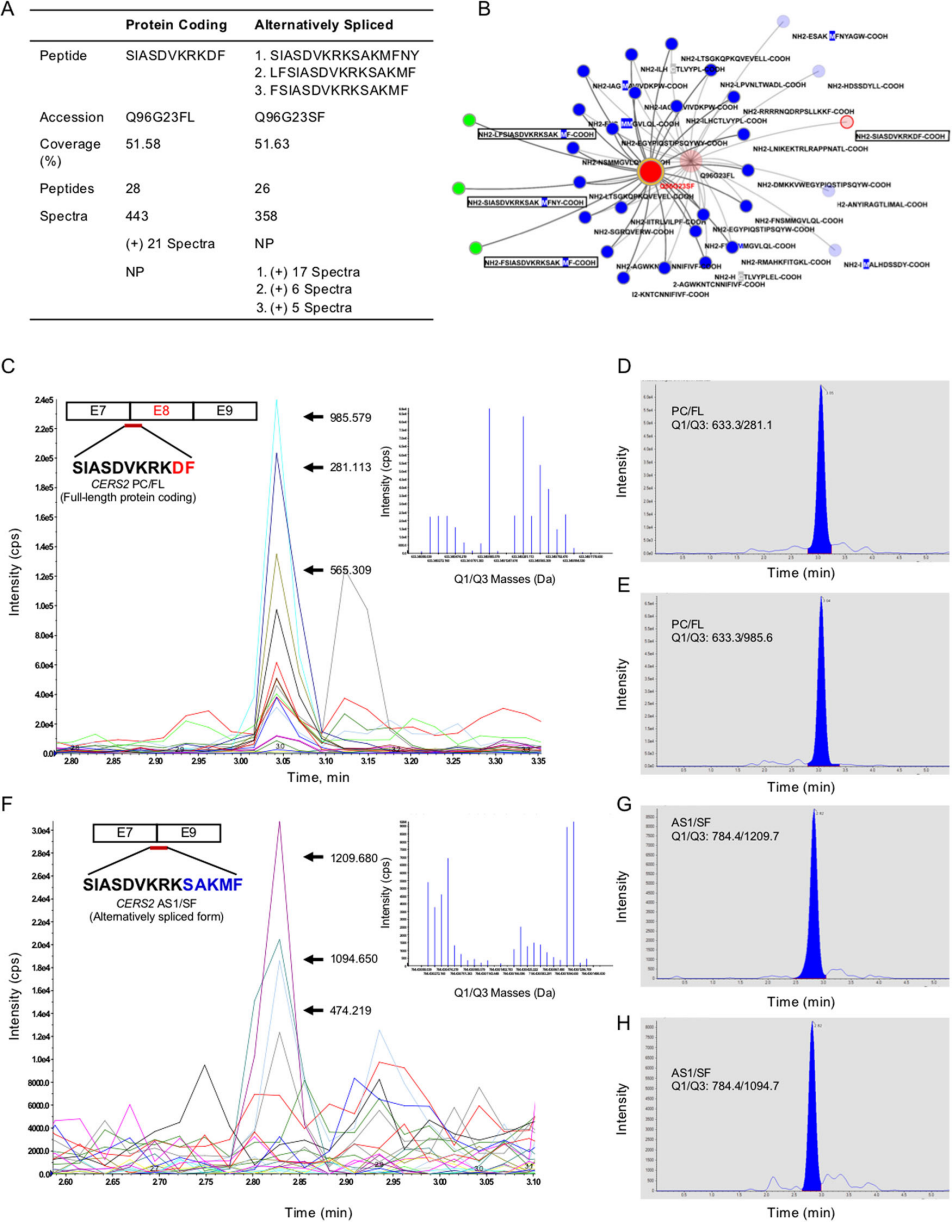
Identification of endogenous proteins translated from full-length PC and alternatively spliced AS1, CERS2 transcripts in BT-474 cells. **A** Table representing the unique peptides corresponding to junction region for full-length (PC/FL, Exon 7–8) and spliced form (AS1/SF, Exon 7–9) of CERS2 proteins (NP = not present). **B** Diagrammatic representation of MS identified peptides corresponding to PC/FL- and AS1/SF-derived proteins. Dark red circle denotes the proteins identified by Peptide Shaker database and blue circles denote all shared peptides identified from the chymotrypsin digested samples in both PC/FL and AS1/SF proteins. Unique peptides covering the junction region for PC/FL or AS1/SF proteins are denoted by light red and green respectively. **C** MRM spectra for PC/FL (Exon 7–8) transcript-derived peptide (SIASDVKRKDF, 196–206 amino acids) showing daughter ions generated by fragmentation of parent ion (633.346 Da *m*/*z*). Inset showing distribution and intensity of daughter ions generated. **D**, **E** Peak area representing abundance of few selected daughter ions generated from parent ion (633.346 Da *m*/*z*) corresponding to PC/FL. **F** MRM spectra for AS1/SF (Exon 7–9) transcript-derived peptide (SIASDVKRKSAKMF, 196–209 amino acids) showing daughter ions generated from the parent ion (784.434 Da *m*/*z*). Inset showing distribution and intensity of daughter ions generated. **G**, **H** Peak area representing abundance of few selected daughter ions as quantified from parent ion (784.434 Da *m*/*z*) corresponding to AS1/SF protein (PC/FL = protein coding/full-length; AS1/SF = alternatively spliced 1/spliced form).

To further validate the existence of stable proteins translated from PC and AS1 transcripts, total endogenous protein from BT-474 cells was immunoprecipitated using anti-CERS2 antibody-conjugated agarose beads. The immunoprecipitated complex was resolved on SDS-PAGE, and the gel region (between 45 and 35 kDa) was excised in form of three gel slices as the immunoblot probed with anti-CERS2 antibody showed the bands in this region (Fig. 3E, Supplementary Figure 15A). The amino acid stretches corresponding to the junction of Exon 7–8 in the protein translated from PC transcript and corresponding to the junction of Exon 7–9 derived from AS1 protein sequence are rich in lysine (K) and arginine (R) residues. Therefore, gel slices corresponding to CERS2 proteins were in-gel digested with chymotrypsin yielding 9–14 amino acid peptides of PC and AS1 protein sequences. Fractions corresponding to three gel slices were analyzed by LC-MS/MS using Information-dependent acquisition (IDA) method and processed by Peptide Shaker software that confirmed ~51% coverage for CERS2 PC/FL (full-length) and CERS2 AS1/SF protein (spliced form) (Fig. 4A, B).

Theoretical chymotrypsin digestion yielded unique peptides spanning the Exon 7–8 junction in PC/FL protein (**SIASDVKR**KD**F**, 196–206 amino acids, 1266 Da) and Exon 7–9 junction (**SIASDVKR**KSAKM**F**, 196–209 amino acids, 1568 Da) in AS1/SF protein. Validation for these peptides was done by LC-MS/MS, multiple reaction monitoring (MRM) mode (Fig. 4C–H). A range of daughter ions were identified (such as b ions: 201.1, 272.2, 359.2, 474.2, 573.3, 701.4, 857.5, 985.6, and 1100.6 and y ions: 994.5, 907.5, 792.5, 693.4, 565.3, 409.2, 281.1, and 166.1) acquired for the [M + 2H]^2+^ doubly charged parent ion (633.346 *m*/*z*) corresponding to the peptide (SIASDVKRKDF, 196–206 amino acids) of Exon 7–8 junction of PC transcript-derived protein (Fig. 4C–E, Supplementary Table 2, Supplementary Figure 15B). We also identified a range of daughter ions (such as b ions: 201.1, 272.2, 359.2, 474.2, 573.3, and 701.4 and y ions: 1367.8, 1296.7, 1209.7, 1094.7, 583.3, 496.3, 425.2, 297.1, and 166.1) for the [M + 2H]^2+^ doubly charged parent ion 784.434 Da *m*/*z* corresponding to the peptide (SIASDVKRKSAKMF, 196–209 amino acids) of Exon 7–9 junction of AS1 transcript-derived protein (Fig. 4F–H, Supplementary Table 2, Supplementary Figure 15C). Therefore, these results confirm that both full-length PC and AS1 endogenous mRNAs are translated.

### Luminal B tumor tissues show a higher expression of alternatively spliced *CERS2*

**Fig. 5:**
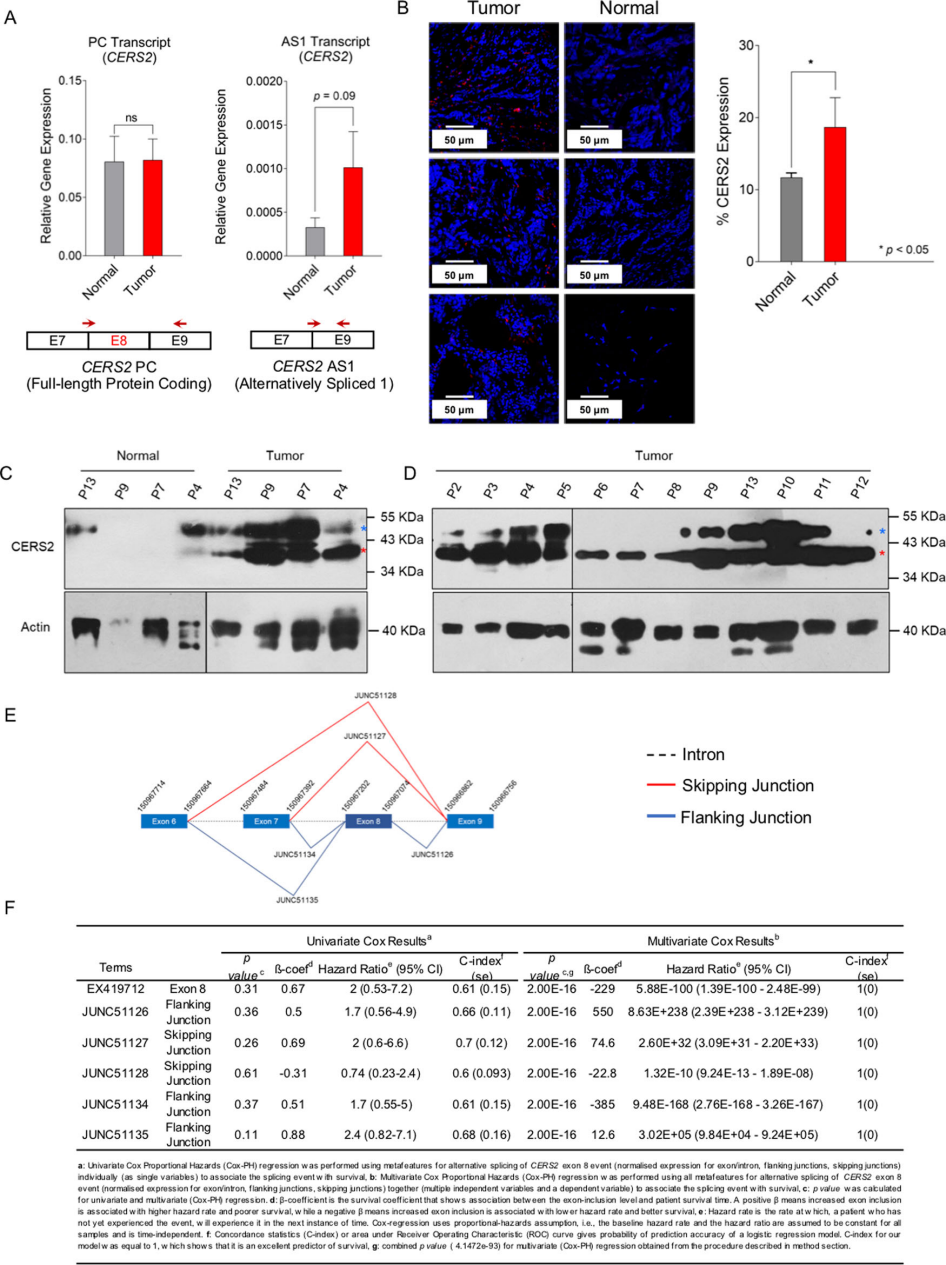
*CERS2* differential isoform expression predict poor prognosis in Luminal B patients. **A** Comparison of differential isoform expression of *CERS2* in tumor (*n* = 10) and matched control (*n* = 10) breast tissues from Luminal B patients using full-length PC and AS1 transcript specific primers show increased AS1 expression in tumor tissue. A pictorial representation of exon/intron position and primer sites are also shown. **B** Immunofluorescence images and quantification of CERS2 (mean ± SD, *n* = 7) show elevated levels in tumor tissue as compared to normal breast tissue sections. **C**, **D** Immunoblot showing the expression of proteins corresponding to PC and AS1 transcripts from 4 pairs of Luminal B tumor and adjoining normal tissue samples (**C**), and 12 patient tumor samples (**D**). Protein corresponding to PC transcript is marked by blue asterisk, and to AS1 transcript by red asterisk. **E**, **F** Survival Analysis on Luminal B patient data (TCGA-BRCA) using univariate and multivariate Cox proportional hazards (Cox-PH) regression. Inclusion of meta-features (exon 8, flanking junctions 51135, 51134, 51126 and skipping junctions 51127, 51128) (**E**), in multivariate analysis significantly affect patient survival (*β* coefficient = −229, *p* < 2e−16, and C-index = 1) (**F**). Data in **A** and **B** were analyzed by two-tailed unpaired Student’s *t*-test.

Next, we validated the predicted AS1 event of *CERS2* in Luminal B tumor tissues excised from Indian patients to strengthen the pathological significance of this event across race and ethnicity. Tumor and adjoining normal breast tissues from 10 Luminal B cancer patients were collected from patients undergoing surgery (Supplementary Table 3). Expression of *CERS2* PC and AS1 transcripts were determined by qRT-PCR in tumor and adjoining normal tissue pairs using event-specific primer pairs (Supplementary Table 1). We observed a ~3-fold increase in the expression of AS1 transcripts in tumor tissues as compared to normal tissues (*p* = 0.09), whereas expression of PC transcript was not markedly different (Fig. 5A). We also quantified the CERS2 expression using immunohistochemistry, and found a ~1.7-fold increase in CERS2 protein expression in tumor tissues as compared to normal tissues (Fig. 5B). On staining the tissue sections with anti-ceramide antibody, we also observed an increase in the level of total ceramides (Supplementary Figure 16). To show that Luminal B tumor tissues express the alternate protein product coded by AS1 transcript, we performed western blot analysis using protein isolated from 4 Luminal B tumor tissue samples and adjoining normal tissue collected from patients (Fig. 5C, Supplementary Table 3). We observed the expression of protein from both PC (marked by blue asterisk) and AS1 (marked by red Asterix) coded transcripts in tumor tissue (Fig. 5C). However, adjoining normal tissue samples show less expression of both PC and AS1 protein products as apparent from a longer exposure of the western blot (Fig. 5C, Supplementary Figure 17). Expression of PC and AS1 protein products in Luminal B tumor tissue were further verified in 12 tumor tissue samples (Fig. 5D). Therefore, these results validate the expression of AS1 protein in tumor tissue along with PC protein. The variation in protein size between cancer cell lines (Fig. 3E) and tumor tissues (Fig. 5C, D) in immunoblot might be due to post-translational modifications as CERS2 protein undergoes glycosylation, and the extent of glycosylation may be different in cell lines and tumor tissue. Earlier reports have shown that *CERS2* expression and ceramide levels are significantly upregulated in malignant breast cancer tumor tissues as compared to benign and normal tissues of ER-positive patients^10,11^. Therefore, the increase in the expression of CERS2 protein in Luminal B tumor tissue, in all probability, arises from full-length PC and elevated AS1 transcripts.

### Exon 8 skipping event in *CERS2* forecasts a poor prognosis

Survival analysis, in general, involves the correlation of differential gene expression with clinicopathological data of patients^44^. However, *CERS2* [*p* = 0.012, log fold change (FC) of 0.94] did not appear as a differentially expressed gene in Luminal B subtype tumors of TCGA-BRCA dataset (Data set 10). Recently, it has been reported that SURVIV (Survival analysis of mRNA Isoform Variation) method using alternative splicing-based survival predictors outperform gene expression-based survival predictors^45^. Therefore, we hypothesized that differential expression of AS1 transcripts of *CERS2* can be a critical contributor to the patient survival over *CERS2* gene expression. Accordingly, we analyzed the effect of AS1 (Exon 8 skipping) of *CERS2* on survival. Exon 8 lies between 150967202 and 150967074 position of *CERS2* on the negative strand of chromosome 1 in humans. Using an in-house script (see “Materials and methods” section), we found three flanking and two skipping junctions associated with Exon 8 (Supplementary Table 4). Upstream of Exon 8, there are two flanking junctions, JUNC51134 (connecting Exon 8 to 7) and JUNC511135 (connecting Exon 6 to 8). There is only JUNC511126 downstream to Exon 8 connecting Exons 8 and 9. There are two skipping junctions to Exon 8, JUNC511127 that connects Exon 7 to 9, and JUNC51128 that connects Exon 6 to 9 (Fig. 5E). So, we considered Exon 8 and its associated terms (meta-features such as flanking junctions and skipping junctions), and performed survival analysis on TCGA-BRCA Luminal B cohort patients (Data set 1) using both univariate and multivariate Cox-PH model. Exon 8 and associated terms, individually, did not show any effect on survival in univariate Cox-PH analysis (*p* = 0.11–0.61) (Fig. 5F). However, multivariate Cox-PH analysis showed that all meta-features (Exon 8 (EX419712), JUNC51127, JUNC51128, JUNC51135, JUNC51134, and JUNC51126) combined together significantly affect patients’ overall survival (Fig. 5F). The Cox regression *p* value for all these terms was found to be highly significant (*p* < 2e−16). Concordance statistics (C-index) gives probability of the prediction accuracy of a logistic regression model^46^. Our model utilizing Exon 8 and its meta-features is a good predictor of patient survival, as confirmed by its C-index (C = 1, s.e. = 0) (Fig. 5F). The result of multivariate Cox analysis shows a decrease in hazard ratio, and suggests that exclusion of Exon 8 in breast cancer patients is a poor prognosis factor (*β*-coefficient = −229) (Fig. 5F).

### Overexpression of *CERS2* (AS1) transcript attenuate levels of very-long-chain ceramides

**Fig. 6:**
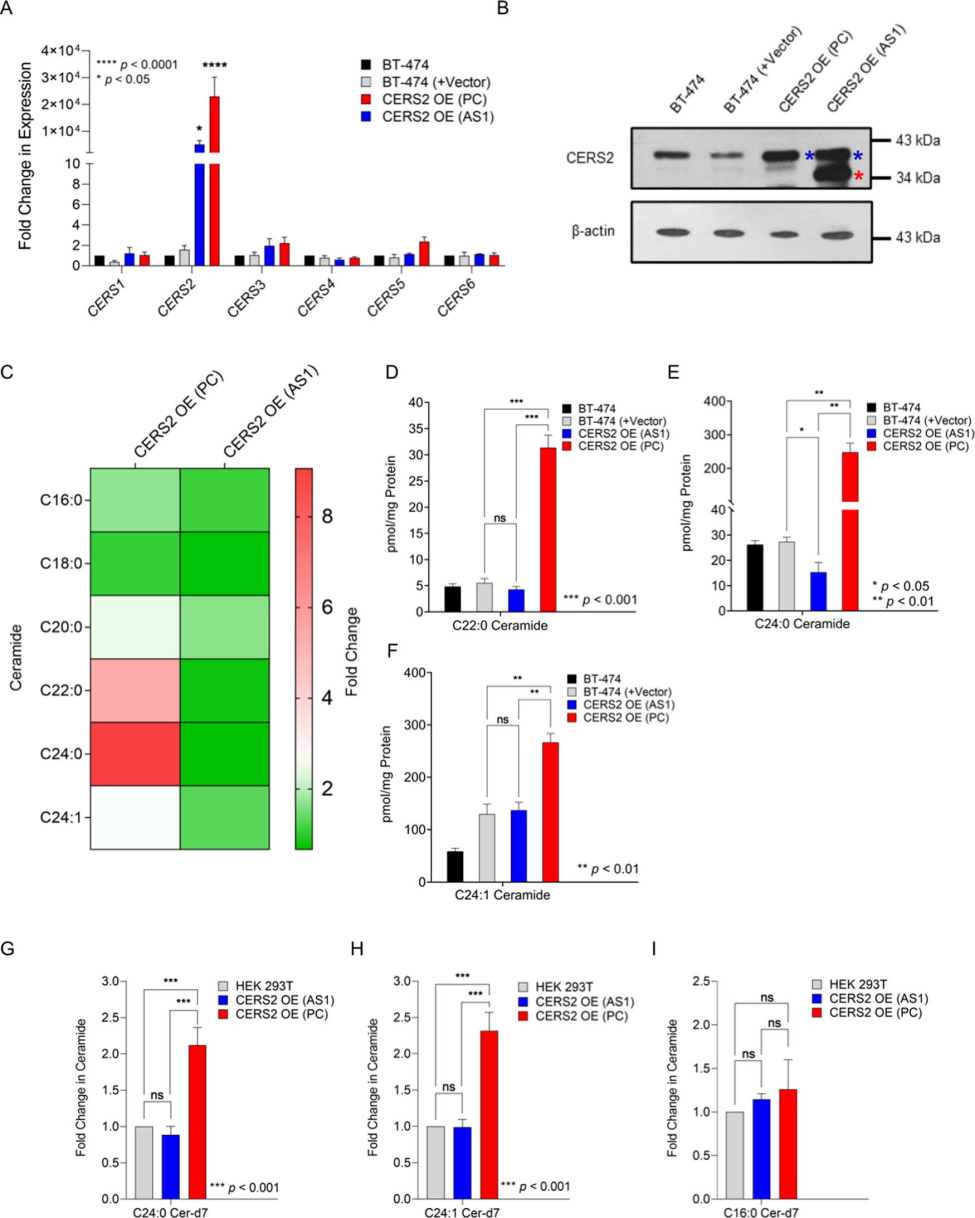
Overexpression of *CERS2* transcript (AS1) decreases expression of very-long-chain ceramides in BT-474 cells. **A** Change in expression of all ceramide synthases (*CERS1-6*) (mean ± SD, *n* = 4) in BT-474 cells transfected with empty vector, BT-474 (+Vector), full-length protein-coding CERS2 OE (PC), and alternatively spliced transcript coding CERS2 OE (AS1) constructs. Expression was normalized to *β*-actin. **B** Immunoblot showing the expression of CERS2 in BT-474, BT-474 (+Vector), CERS2 OE (PC), and CERS2 OE (AS1) cells by western blot analysis. Protein corresponding to PC transcript is marked by blue asterisk, and to AS1 transcript by red asterisk. **C** Heat map showing the average fold change in levels of ceramides (C16:0, C18:0, C20:0, C22:0, C24:0, and C24:1) in BT-474 cells transfected with CERS2 OE (PC) and CERS2 OE (AS1) with respect to BT-474 cells. **D**–**F** Absolute quantification of ceramides (mean ± SD, *n* = 4) show significant decrease in C22:0 (**D**), C24:0 (**E**), and C24:1 (**F**) ceramides in CERS2 OE (AS1) cells as compared to CERS2 OE (PC) cells. **G**–**I** Fold change (mean ± SD, *n* = 4) in deuterium-labeled ceramides (pmol/mg protein) in HEK 293T cells transfected with CERS2 OE (PC) and CERS2 OE (AS1) constructs using HEK 293T cells as a control shows significant decrease in C24:0 Ceramide (Cer)-d7 (**G**) and C24:1 Cer-d7 (**H**) ceramides with no significant change in C16:0 Cer-d7 (**I**) ceramides in CERS2 OE (AS1) cells as compared to CERS2 OE (PC) cells. Data in **A**, **D**–**I** were analyzed using ANOVA test.

To functionally validate the effect of AS1 transcripts on CERS2 catalytic activity, we overexpressed PC and AS1 transcript-encoding cDNAs in BT-474 cells by transfecting the cells with only vector or *CERS2* OE (PC) or *CERS2* OE (AS1) constructs. *CERS2* OE (PC) construct allows the overexpression of protein-coding transcript whereas *CERS2* OE (AS1) construct helps in overexpression of AS1 transcript. Overexpression of PC and AS1 transcripts led to the selective increase in the corresponding *CERS2* transcript levels (Fig. 6A). We did not observe any change in expression of other *CERSs* on overexpression of *CERS2* PC and AS1 transcripts (Fig. 6A). Immunoblot analysis confirmed the overexpression of protein products of *CERS2* OE (PC) or *CERS2* OE (AS1) constructs after 36 h of transfection (Fig. 6B), where we observed the protein band translated from AS1 transcripts in *CERS2* OE (AS1) overexpressed BT-474 cells (Fig. 6B). The endogenous CERS2 PC gets also increased in the CERS2 OE (AS1) overexpressing cells, that may be due to a recalibration effort by the cells. CERS2 is known to interact and form dimer with other CERS enzymes like CERS5 and CERS6 (ref. ^47^). We, therefore, checked for any alterations in expression of CERS1, CERS5, and CERS6, and did not observe any appreciable change due to overexpression of CERS2 (Supplementary Figure 18).

Quantitative estimation of ceramides from BT-474, BT-474 (+Vector), *CERS2* OE (PC), and *CERS2* OE (AS1) transfected cells was performed using liquid-chromatography mass spectrometry (LC-MS/MS) in multiple reaction monitoring (MRM) mode. Heat map shows average fold change in ceramide levels (C16:0, C18:0, C20:0, C22:0, C24:0, and C24:1) in CERS2 OE (PC) and CERS2 OE (AS1) overexpressed BT-474 cells compared to BT-474 (+Vector) cells (Fig. 6C, Data set 11). Quantitation of ceramide species showed ~7.4- (*p* < 0.001), ~16.29- (*p* < 0.01), and ~1.95 (*p* < 0.01)-fold decrease in expression of C22:0, C24:0, and C24:1 ceramides in CERS2 OE (AS1) transfected cells compared to CERS2 OE (PC) transfected cells (Figs. 6D–F). There were no significant alterations in C16:0, C18:0, and C20:0 ceramide species between CERS2 OE (AS1) and CERS2 OE (PC) overexpressed BT-474 cells (Supplementary Figure 19). Therefore, these results suggest that unlike CERS2 OE (PC) cells, CERS2 OE (AS1) BT-474 cells are unable to increase the levels of very-long-chain ceramides.

To confirm whether CERS2 OE (AS1) cells lack catalytic specificity for synthesizing the very-long-chain ceramides, we performed in vitro CERS2 activity assay. We overexpressed *CERS2* OE (PC) and *CERS2* OE (AS1) transcript-encoding cDNAs in HEK 293T cells, and prepared membrane fractions. After normalizing the protein content, equivalent quantity of membrane preparations were incubated with deuterium-labeled sphingosine and different acyl chain-length CoAs (C16:0-CoA, C24:0-CoA, and C24:1-CoA)^48^. Deuterium-labeled ceramides generated (C16:0 Cer-d7, C24:0 Cer-d7, and C24:1 Cer-d7) were quantified by LC-MS/MS. There was ~2.1-fold (*p* < 0.05) increase in C24:0 Cer-d7 levels, and ~2.3-fold (*p* < 0.05) increase in C24:1 Cer-d7 levels in *CERS2* OE (PC) transfected cells compared to *CERS2* OE (AS1) transfected BT-474 cells (Fig. 6G, H). However, there was no significant difference in C16:0 Cer-d7 levels between PC and AS1 overexpressed cells (Fig. 6I). Therefore, these results validate the specific catalytic activity of CERS2 for very-long-chain ceramides in CERS2 OE (PC) cells than CERS2 OE (AS1) BT-474 cells.

### Overexpression of *CERS2* (AS1) transcript fails to suppress proliferation and migration of BT-474 cells as effectively as PC transcript

**Fig. 7:**
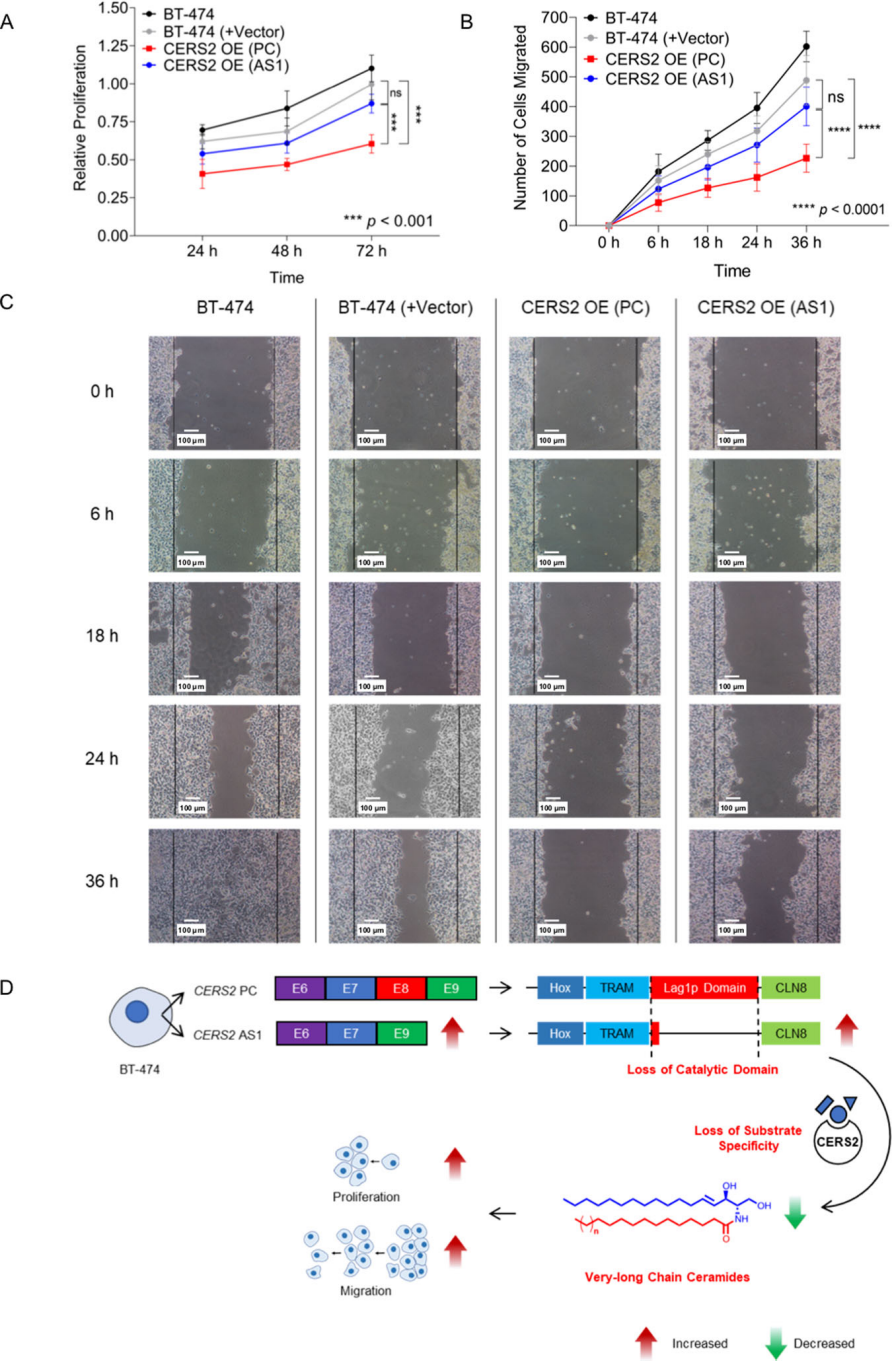
Overexpression of *CERS2* transcript (AS1) is unable to suppress proliferation and migration of BT-474 cells. **A**, **B** Change in proliferation (**A**) (mean ± SD, *n* = 5) and number of migrating cells (**B**) (mean ± SD, *n* = 5) shows that overexpression of CERS2 OE (AS1) in BT-474 cells significantly enhances cell proliferation estimated by MTT assay (**A**) and the number of migrating cells in scratch wound assay (**B**) compared to overexpression of CERS2 OE (PC) cells. **C** Representative images of wound closure at 0, 6, 18, 24, and 36 h in BT-474, BT-474 (+Vector), CERS2 OE (AS1), and CERS2 OE (PC) cells. **D** Model depicting how a post-transcriptional regulatory mechanism affects sphingolipid metabolism in Luminal B subtype-specific cancer cells by downregulating the synthesis of very-long-chain ceramides that facilitate tumor progression by increased cell proliferation and migration. Data in **A**, **B** were analyzed using two-way ANOVA.

In addition to its fundamental function as a very-long-chain *ceramide synthase*, *CERS2* has been shown to inhibit tumor invasion and metastasis^49–51^. Overexpression of CERS2 induces significant suppression in proliferation and migration of breast, prostate, and bladder cancer cells^52^. To discern the role of Lag1p domain encoded by *CERS2* Exon 8 on proliferation of Luminal B-specific BT-474 cells, we compared the proliferation profiles of *CERS2* OE (PC) BT-474 cells with BT-474, BT-474 (+Vector), and *CERS2* OE (AS1) BT-474 cells using MTT assay. CERS2 OE (PC) BT-474 cells showed a ~1.6-fold (*p* < 0.001) decrease in proliferation compared to BT-474 (+Vector) cells, and ~1.4-fold (*p* < 0.001) reduction in proliferation compared to CERS2 OE (AS1) cells after 72 h. In contrast, CERS2 OE (AS1) cells did not show any significant decrease in proliferation compared to BT-474 (+Vector) cells (Fig. 7A). Therefore, these results suggest that AS1 transcript overexpressing cells have higher proliferation rates over PC transcript overexpressed cells, and overexpression of PC transcripts inhibit the proliferation of Luminal B subtype-specific cancer cells.

To elucidate the role of Lag1p domain on cell migration, we quantified the number of migrating cells using scratch wound assay for 36 h after overexpression of PC and AS1 transcripts, and compared with BT-474 and BT-474 (+Vector) cells. *CERS2* OE (AS1) cells showed ~1.77-fold (*p* < 0.0001) increase in number of migrating cells compared to CERS2 OE (PC) cells (Fig. 7B, C). CERS2 OE (PC) cells showed ~2.15-fold decrease (*p* < 0.00001) in number of migrating cells compared to BT-474 (+Vector) cells whereas CERS2 OE (AS1) cells did not show any significant change in number of migrated cells compared to BT-474 (+Vector) cells (Fig. 7B, C). There was >55% increase (*p* < 0.0001) in number of CERS2 OE (AS1) migrating cells as compared to CERS2 OE (PC) cells (Fig. 7B, C). Therefore, these results suggest that overexpression of PC transcript and increased levels of very-long-chain ceramides help in reduced migration of Luminal B-specific BT-474 cells. In contrast, reduced levels of very-long-chain ceramides in CERS2 OE (AS1) cells may be the reason that they cannot supress the migration of BT-474 cells similar to PC overexpressing cells, in spite of showing a comparable expression of PC-derived protein product (Figs. 6B, 7D).

To confirm that the difference in proliferation and migration between CERS2 OE (PC) and CERS2 OE (AS1) cells is not due to cell death, we performed cell death assay by Annexin-FITC/Propidium Iodide assay. Overexpression of CERS2 OE (PC) and CERS2 OE (AS1) transcripts induced some apoptosis in BT-474 cells. There was only a ~5% increase in apoptosis in CERS2 OE (PC) overexpressed cells compared to CERS2 OE (AS1) cells (Supplementary Figure 20), whereas we observed 30% decrease in proliferation and >55% decrease in migration in CERS2 OE (PC) cells compared to CERS2 OE (AS1) cells. Therefore, these results reveal a significant contribution of skipping of Exon 8 in CERS2 towards cancer cell proliferation and migration in Luminal B subtype-specific cancer cells.

## Discussion

Genome-wide approaches reveal that cancer development and progression involves massive changes in AS events affecting the tumor proteome. This is evident from the large repertoire of subtype-specific CE and IR events scored from our study on TCGA-BRCA RNA-Seq data. Events specific to sphingolipid genes predict that AS contributes distinctly to reprogramming of sphingolipids in breast tumors of different subtypes. This may be the possible link between high levels of sphingolipids like ceramides and sphingomyelins in tumor tissues, contrary to their antiapoptotic role established in in vitro and in vivo studies. Identification of unique and common AS events in ceramide synthase genes suggest that AS may be responsible for chain-length-specific accumulation of ceramides in subtype-specific breast tumor tissues. The increase in ceramides in response to AS events may therefore contribute to the lipid metabolic reprogramming required during tumor progression^53,54^.

Validation of one of the predicted AS transcripts for *CERS2* in Luminal B-specific BT-474 cells showed the expression of AS1 transcript lacking Exon 8 along with full-length PC transcript. Both transcripts get translated endogenously as observed by the polysome assay and LC-MS/MS-based MRM identification of corresponding peptides spanning the junction between Exon 7–9 for AS1 and Exon 7–8 for PC-derived proteins. Lag1p domain is an essential part of TLC domain of CERSs that helps in selecting different acyl-CoAs to generate chain-length-specific ceramides. Recently, it has been shown that a 11 residue region (amino acid 299–309) in the last luminal loop between the putative transmembrane domains 5 and 6 confers acyl chain substrate specificity^55^. However, acyl-CoA specificity assay using two sets of chimeric proteins, where 11 residue region was swapped between CERS2 and CERS5 showed that CERS5^(299-309 CERS2)^ can utilize very-long-chain acyl-CoAs but CERS2^(299-309 CERS5)^ did not show any activity towards both short and long-chain acyl-CoAs. Therefore, apart from the 11 residue domain, there may be other determinants like Lag1p domain assigning the activity and substrate chain specificity to CERS enzymes. Our study shows that *CERS2* OE (AS1) lacking almost the entire Lag1p domain loses its specificity in tethering the very-long-chain acyl-CoAs to the sphingosine backbone compared to *CERS2* OE (PC) that have an intact Lag1p domain. Therefore, in BT-474 cells, CERS2 AS1 protein probably does not disrupt the activity of the PC-derived protein but disrupts the balance between very-long-chain ceramides and other short/long-chain ceramides. Thus, this post-transcriptional regulatory mechanism possibly disturbs the relative balance of different chain-length-specific “ceramide pools” in tumor tissues^56^.

A balance of short-chain (C16:0), long-chain (C18:0, C20:0), and very-long-chain (CC22:0, C24:0, C24:1) ceramides is critical for determining the fate of cancer cells depending on the cancer type^57^. Overexpression of *CERS4* and *CERS6* in breast and colon cancer cells, leading to an increase in short (C16:0) and long-chain ceramides (C18:0 and C20:0), inhibited cell proliferation and increased apoptosis^58^. At the same time, accumulation of long-chain C18:0 ceramides in human head and neck squamous cell carcinoma suppressed the proliferation of cancer cells, whereas short-chain C16:0 ceramides induced proliferation^4^. C16:0 ceramides have been shown to play a pivotal role in cell migration during epithelial to mesenchymal transition in different cancer cell lines^59^. In contrast, overexpression of CERS2, that synthesizes very-long-chain ceramides, has been demonstrated to inhibit cancer cell proliferation and migration in prostate, breast, and bladder cancers^60,61^. Luminal B-specific BT-474 cells overexpressing PC transcript of *CERS2* showed a significant decrease in cell proliferation and migration due to an increase in levels of very-long-chain ceramides. In contrast, overexpression of alternatively spliced CERS2 OE (AS1) did not alter the migration and proliferation of BT-474 cells significantly, reflecting the effect of a disbalance in the level of very-long-chain ceramides. Therefore, disequilibrium between short, long, and very-long-chain ceramides and the balance between AS transcripts of critical genes regulating the ceramide metabolism adds on to the myriad of factors that lead to cancer progression.

Luminal B subtype tumors are marked with increased expression of proliferation-related genes, more aggressive clinical behavior, and significantly unfavorable prognosis as compared to Luminal A subtype^62–64^. In spite of better prognosis after treatment, there is a higher proportion of local recurrence as well as a persisting risk of metastasis in Luminal B patients in comparison to non-Luminal cancers^65^. Most commonly-used survival methods for retrospective studies of clinical data undermine the contributions of differential AS of genes. SURVIV, a statistical method, identifies and associates mRNA isoform variation with survival time in both censored and uncensored large-scale RNA-Seq data. Exon inclusion events were found to be significantly correlated with survival time in TCGA ductal carcinoma patients when SURVIV was used. Combined effect of Exon 8 skipping event and associated meta-features of *CERS2* in Luminal B patients from TCGA-BRCA cohort shows that exclusion of Exon 8 of *CERS2* predicts poor survival. Therefore, contribution of post-transcriptional regulatory events needs to be considered for better interpretation of clinical outcome of cancer patients. It is prudent to mention that our data validated for Luminal B subtype will be further strengthened by future studies in Indian patient tissues of other subtypes.

In summary, our results show that AS of sphingolipid genes provide a unique signature for breast cancer subtypes. Luminal B-specific AS of *CERS2* reduces the levels of very-long-chain ceramides that allows enhanced proliferation and migration of cancer cells, and a poor prognosis outcome for patient survival. Therefore, identification of post-transcriptional regulatory mechanisms responsible for sphingolipid metabolism can provide new therapeutic strategies for combating tumor progression.

## Materials and methods

### Classification of breast cancer subtypes

The clinical data and bam files for TCGA-BRCA RNA-Seq data (817 samples) were downloaded from the GDC portal (project number #12293 and #12397), NIH, after due approval from database of Genotype and Phenotype (dbGAP). The samples belonging to the broad category of Invasive Ductal Carcinoma were classified into intrinsic subtypes: Luminal A (ER^+^, PR^+/−^, HER2^−^), Luminal B (ER^+^, PR^+/−^, HER2^+^), HER2^+^ (ER^−^, PR^−^, HER2^+^), and Basal (ER^−^, PR^−^, HER2^−^) by their PAM50 status as well as the hormone receptor status (Data set S1).

### Alternative splicing (AS) analysis

Differential alternative splicing (AS) in Luminal A, Luminal B, HER2^+^, and Basal tumor tissues in comparison to normal-like tissues were identified by Exon Pointer (EP) and Intron Pointer (IP) algorithm as per published protocol using in-house pipeline^32^.

### GO enrichment analysis

Enrichment analysis of all the genes predicted by AS analysis for CE and IR events was performed using the Database for Annotation, Visualization, and Integrated Discovery v6.8 (DAVID). All default parameters were used, and cut off was kept at *p*-value ≤ 0.05 (Fisher’s exact test) and/or false discovery rate (FDR) of ≤15.0. The functional association was done using Biological processes (GO_BP_Direct), KEGG Pathways, and Protein domains. The raw data and values are provided in Data sets 4–7.

### Survival analysis

We have designed and used a novel strategy to predict survival analysis using exon/intron level RNA expression counts combined with associated meta-features in a given splicing condition, i.e., flanking junction and skipping junction to the respective exons/introns. Since we are interested to find the cumulative and individual effect of two or more terms (exons and junctions) in a gene, we applied multivariate Cox regression model and univariate Cox regression model, respectively^66,67^. The resulted term with multivariate Cox-PH and univariate analysis with *p*-value <0.05 were considered as significantly affecting the survival. Multivariate Cox-PH analysis gives individual *p*-value for exon/intron and its associated terms (flanking and skipping junction). To find the combined effect of given exon/intron and associated terms, these *p*-values can be summarized using Irwin–Hall method as follows for a cassette event:

1 $$x_e = \mathop {\sum }\limits_{k \in H_e} pvalues_k$$

where, (*k* ∈ *H*_*e*_) denotes the set of terms (exon *e*, flanking junctions, and skipping junctions) where flanking junctions are expressed with exon e when included into a transcript for a given gene k, and skipping junctions are expressed when exon e is not included into transcript. For an intron retention event, (*k* ∈ *H*_*e*_) will include intron *e* and skipping junction. For the event described in results (Fig. 4C), the Eq. (2) will be as follows;

2 $$x_{exon8} = \mathop {\sum}\limits_{\scriptstyle k \in \left\{ {exon8,JUNC51134,} \right.\hfill \atop\\ {\scriptstyle JUNC51135,JUNC51126,\hfill \atop\\ \scriptstyle \left. {JUNC51127,JUNC51128} \right\}\hfill }} {pvalues_k}$$

In case the null hypothesis is true, the sum of uniform distribution will behave as uniform distribution according to Irwin–Hall method^68^. Given the X statistic is smaller than one, the corresponding probability distribution function (piecewise polynomial function) would be;

3 $$f_{X^{(x)}} = \frac{1}{{(n - 1)}}.x^{(n - 1)},\;x \le 1$$

where *x* is the summation of the *p*-values mentioned in Eq. (2). The probability to obtain a combined *p*-value is given by:

4 $$p-{\mathrm{value}}^{{\mathrm{combine}}} = 2{\int}_0^{x_e} {f_x\left( x \right)dx = 2\frac{{x_e}}{{n!}}}$$

where *p*-value^combine^ is the summarized *p*-value given for exon or intron along with associated terms upon significantly affecting the survival.

### Cell culture

Human breast cancer cell lines BT-474, MDA-MB-453, MDA-MB-231, and human embryonic kidney cells (HEK 293T) were grown and maintained in Dulbecco modified Eagle medium (DMEM) (Sigma, D5648) supplemented with 10% (v/v) fetal bovine serum (FBS) (Gibco, 10270) and 1% penicillin–streptomycin (Hyclone, 113-98-43810-74-0). Non-tumorigenic mammary epithelial (MCF-10A) cells were grown and maintained in complete mammary epithelial basal medium (MEBM) (Lonza, CC-3151). All cell lines were maintained at 37 °C, 5% CO_2_, and 95% humidity in a CO_2_ incubator.

### Cloning

The full-length *CERS2* (PC) (1143 bps) and alternatively spliced form of *CERS2* (AS1) (1014 bps) were cloned in pBBL-FLAG vector (BioBharti LifeScience Pvt Ltd) using Xho1 (NEB, R0104S) and Hind III (NEB, R0146S) restriction enzymes. For amplification, the recombinant plasmids were transformed in *Escherichia coli* (DH5α) competent cells, and the positive colonies were selected using kanamycin (GoldBiocom, 25389-94-0). The *CERS2* OE (PC) and *CERS2* OE (AS1) plasmids were extracted using the plasmid extraction kit (Qiagen, 12143).

### Transfection protocol

BT-474 (or HEK 293T) cells (~2.5 × 10^5^/well) were seeded in a six-well plate in complete DMEM media. After 24 h, media was carefully removed from each well, and cells were washed with DPBS (Sigma, D5652). Cells were then incubated with a transfection mix comprising 1000 µL Opti-MEM (Gibco, 31985070), 5 µL Lipofectamine (Invitrogen, 11668019), and 2 μg of plasmid PBBL-FLAG vector (control vector) or *CERS2 OE* (PC) or *CERS2 OE* (AS1) plasmid in respective wells. After 6 h of transfection, media was replaced with antibiotic-free DMEM media containing 10% FBS, and cells were incubated for 24–48 h. Media was removed, and cells were rinsed carefully with 1X DPBS. To collect the cells for RNA isolation, 1 mL of RNAiso Plus (DSS Takara, 9109) was added to each well, and incubated for 5 min. The cells were then aspirated slowly using a pipette. To collect the cells for protein and lipid isolation, cells were scraped from the wells. The cell pellets were rinsed with DPBS by centrifugation at 2500 rpm for 5 min for three times. Pellets were then air-dried and stored at −80 °C.

### Quantitative real-time PCR

Frozen cell pellets in RNAiso Plus were homogenized in Trizol (Qiagen, 79306). RNA was purified by phenol:chloroform:isoamyl alcohol extraction followed by ethanol precipitation. RNA concentration was determined using NanoDrop 2000 (ThermoScientific, USA). The integrity and quality of ribosomal 28S and 18S were determined on the agarose gel. Any traces of genomic DNA were removed by DNase (Invitrogen, Am2238) treatment for 30 min at 37 °C followed by heat inactivation using 50 mM EDTA (Sigma, E5134). cDNA synthesis and real-time PCR were done as described previously^27^. Relative quantitation of gene expression was done using *β*-actin as the endogenous reference gene for normalization. All primer sequences used for RT-PCR are listed in Supplementary Table 1.

### Western blot

Cell pellets were lysed in RIPA lysis buffer (50 mM Tris buffer, pH 7.4, 100 mM NaF; 120 mM NaCl, 0.5% NP-40, 100 μM Na_3_VO_4_) with 1X protease inhibitor cocktail (Fermentas, R1329). Patient tumor and adjoining normal tissue were also homogenised in RIPA lysis buffer with 1X protease inhibitor cocktail. The protein was collected by centrifugation at 13,000 rpm for 10 min at 4 °C, and quantified using bicinchoninic acid (BCA) protein estimation kit (ThermoScientific, 23227). Using SDS-PAGE, ~20 μg of cell lysates or ~25 μg of patient tissue samples were resolved on 10 or 15% gel for separation, and transferred to the PVDF membrane (Merck, IPVH00010). Immunostaining was done using CERS1 (1:5000 dilution) (Abnova, H00010715-A01), CERS2 (1:400 dilution) (SantaCruz, sc-390745), CERS5 (1:2000 dilution) (Abcam, ab73289), CERS6 (1:5000 dilution) (Abnova, H00253782-M01), or *β*-actin (1:10,000 dilution) (Sigma, A5441) by overnight incubation at 4 °C in 5% BSA in TBST solution (1X TBS; 0.1% Tween 20). After washing, the blots were incubated with goat anti-mouse IgG (L + H) secondary antibody (1:10,000 dilution) (Abcam, ab6789) or anti-rabbit IgG HRP secondary antibody (1:10,000 dilution) (SantaCruz, sc-2004) for 1 h. Exposed X-ray sheets (Carestream, 6568307) were developed using Immobilon Western Chemiluminescent HRP (Merck Millipore, WBKLS0500).

### Polysome profiling

BT-474 cells were grown in a 10 cm petri dish to ~80% confluency. Cells were incubated with cycloheximide (100 µg/mL) at 37 °C for 15 min, and washed with ice-cold PBS containing 100 µg/mL cycloheximide. Plates were frozen in a shallow bath of liquid nitrogen. Cells were lysed in 400 µL of ice-cold lysis buffer (20 mM Tris-Cl, pH 7.4; 150 mM NaCl, 5 mM MgCl_2_, 1 mM DTT, 100 µg/mL cycloheximide, 1% Triton X-100, 0.2% sodium deoxycholate, 1 mM CaCl_2_, 2.5 mM PMSF, 1X complete protease inhibitors (Roche, CO-RO), 40 U/mL SUPERase.In^TM^ (ThermoScientific, AM2694)) by passing through a 26G needle 10 times. After clearing by centrifugation, lysate was loaded on a 10–50% sucrose gradient (20 mM Tris-Cl pH 7.4, 150 mm NaCl, 5 mM MgCl_2_, 1 mM DTT, 100 µg/mL cycloheximide, sucrose 10–50%), and centrifuged in a SW41Ti rotor (Beckman) at 36,000 rpm for 2 h and 10 min. The gradient was fractionated, and absorbance at 260 nm was recorded using a gradient station (Biocomp Instruments, Fredericton, Canada). RNA was isolated from the fractions, and subjected to RT-PCR using primers specific for full-length protein-coding and alternatively spliced transcripts (Supplementary Table 1).

### Immunoprecipitation and mass spectrometry

BT-474 cells were lysed in RIPA lysis buffer, and total protein was isolated and quantified using BCA protein estimation kit. Protein equivalent to 1 mg (in 1000 μL lysate) was mixed with 20 μL (equivalent to 10 μg) of LASS2/CERS2 antibody-conjugated to agarose beads (SantaCruz, sc-390745 AC) at 4 °C for overnight. Bound protein complexes were centrifuged, washed two times with 500 μL buffer, and the supernatant containing unbound protein was discarded. Anti-CERS2 antibody-immunoprecipitated complexes were eluted in 30 μL SDS-PAGE Laemmli sample buffer. Protein complexes were resolved on 15% SDS-PAGE, and stained with 0.1% Coomassie stain (Sigma, 27816).

Gel slices corresponding to CERS2 bands (PC and AS1 translated proteins) from 45 to 35 kDa were cut out, and de-stained using 50 mM ammonium bicarbonate and 50% acetonitrile (Honeywell, 34967). Gel slices were hydrated with 10 mM DTT, alkylated using 20 mM iodoacetamide (Sigma,144-48-9), and dehydrated using 100% acetonitrile before digestion. Gel pieces were then treated with chymotrypsin (pH 8) (Merck, 11418467001), and incubated at 37 °C for 18 h. The peptides were extracted two times by addition of 60% acetonitrile and 0.1% formic acid (Fluka, 56302-50 ML) followed by ultrasonication. Peptides were collected, vacuum dried, and resuspended in 15 μL of solvent A (98% water, 2% acetonitrile, and 0.1% formic acid). Peptides were analyzed by liquid-chromatography–mass spectrometry (LC−MS/MS) (Sciex 5600^+^ Triple-TOF mass spectrometer), coupled with ChromXP reversed-phase 3 μm C18-CL trap column (350 μm × 0.5 mm, 120 Å, Eksigent, ABSciex, 5016752) and nanoViper C18 separation column (75 μm × 250 mm, 3 μm, 100 Å; Acclaim Pep Map, ThermoScientific, 164569) in Eksigent nanoLC (Ultra 2D plus) system. The binary mobile solvent system used was solvent A (2% acetonitrile, 0.1% formic acid in water) and solvent B (98% acetonitrile, 2% water, 0.1% formic acid). Peptides were separated at a flow rate of 250 nL/min in a 16 min gradient with a total run time of 35 min. MS data of each condition were acquired in IDA (information-dependent acquisition) with high sensitivity mode. Each cycle consisted of 250 and 100 ms accumulation time for MS1 (*m*/*z* 400−1250 Da) and MS/MS (100–1500 *m*/*z*) scans, respectively.

The MS/MS spectrum raw files (.wiff) were converted in .mgf format, and a search output file was created using the “SearchGUI 3.3.16” platform coupled with Andromeda search engine. Data were searched against the database that consisted of full-length protein-coding (PC/FL) and spliced form (AS1/SF1) of CERS2 protein sequence (Supplementary Figure 7). The search parameters for identification of interested peptide sequences of CERS2 protein from the digested samples were as follows: (a) chymotrypsin as a specific proteolytic enzyme (with up to two missed cleavages), (b) peptide mass error tolerance of 100 ppm, (c) fragment mass error tolerance of 0.50 Da, (d) precursor charge range 2–4, and (e) carbamidomethylation of cysteine (+57.02 Da), oxidation of methionine (+15.99 Da), deamination of NQ (+0.98) as variable modifications. The data analysis and post-processing were done by Peptide Shaker 1.16.42 version using the entire protein sequence of PC/FL and AS1/SF for the search.

Protein sequences corresponding to PC and AS1 protein product were theoretically digested by EXPASY Peptide Mass tool using chymotrypsin enzyme. Detection of peptide masses as [M + 2H]^2+^ identified PC-derived peptide spanning the junction of Exon 7–8 with a sequence of SIASDVKRKDF (196–206) having molecular weight of 633.346 Da. In contrast, theoretical digestion of AS1 protein product provides a unique AS1-derived peptide spanning the junction of Exon 7–9 with sequence of SIASDVKRKSAKMF (196–209) having molecular weight of 784.434 Da. The junction peptide sequences were analyzed in silico in PeakView^®^ (version 2.2; SCIEX, USA) software to identify theoretical daughter ions (b and y ions *m*/*z*, Supplementary Table 2) generated from both PC/FL and AS1/SF-derived parent ions as mentioned above to construct a multiple reaction monitoring (MRM) method. The collision energy for fragmentation was calculated based on the straight line equation, CE = [Slope] × (*m*/*z*) + [intercept], where slope = 0.0625 and intercept = −3 were used for doubly charge peptides as per the suggestion given by the manufacturer. The peptides (Exon 7–8 junction in PC and Exon 7–9 junction in AS1) were identified by MRM scan on a triple quadrupole/linear ion trap mass spectrometer (6500 QTRAP, SCIEX, USA) coupled to a LC system. A Kinetex^®^ C18, 2.1 × 50 mm column (Phenomenex®, 00B-4601-AN) with a particle size of 5.0 μm was used with 5 μL injection volume, and 40 °C oven temperature. The total run time optimized was 10 min. Solvents used were Solvent A (100% water with 0.1% formic acid) and solvent B (100% acetonitrile with 0.1% formic acid) at 0.3 mL/min flow rate. The gradient program used for separation was 5% B–50% B from 0 to 6 min, 50% B–80% B from 6 to 8 min, holding 80% B up to 8.5 min, 80% B–5% B from 8.5 to 9.0 min and keeping 5% B till 10 min. Data analysis and quantification were performed by Analyst^®^ (version 1.7; SCIEX, USA) and MultiQuant™ software (version 3.0.2; SCIEX, USA).

### Collection of patient tumor tissues

Patient tumor and adjoining normal breast tissue were collected from operable breast cancer Luminal B patients (Stages I, II, IIIa) undergoing treatment at Dr. B. R. Ambedkar Institute-Rotary Cancer Hospital (BRA-IRCH), All India Institute of Medical Sciences (AIIMs), New Delhi, and from biorepository of Rajiv Gandhi Cancer Institute and Research Center (RGCIRC) Delhi after due Ethical clearance from AIIMS (IEC-332/01.07.2016), RGCIRC (RGCIRC/IRB/276/2019, Res/BR/TRB-20/2020/70), and Amity University Haryana (IEC-AIISH/AUH/2016-1). Informed consent was taken from all patients before acquiring samples.

Inclusion criteria include the women patients of all age (18–85 years) of any socioeconomic status who have given consent for tissue collection, patients with operable breast cancers (Stages I, II, IIIa) who will undergo adjuvant therapy, and the patients with estrogen receptor-positive (ER^+^), progesterone (PR^+^), and HER2^+^ status. Exclusion criteria include patients who are undergoing neo-adjuvant chemotherapy, and patients who are not fit to undergo surgery. Details of patients and tumor signature are mentioned in Supplementary Table 3.

### RNA isolation and quantitative real-time PCR from tumor tissues

Patient tissue samples (~20 mg) in Allprotect Tissue Reagent (Qiagen, 76405) were homogenized in QIAzol Lysis reagent (Qiagen, 74804) using mortar pestle. After homogenization, the samples were incubated at room temperature for 5 min. Phase separation was done by addition of chloroform followed by centrifugation. The aqueous layer was collected, and ethanol was added. The samples were then applied to Qiagen RNeasy spin column (Qiagen, 74804), and centrifuged, washed, and eluted using 30 µL of RNase-free water. The RNA concentration was quantified using NanoDrop 2000 (ThermoScientific, USA) spectrophotometer at OD_260_, and purity was determined by OD_260/280_ ratio. cDNA synthesis and real-time PCR were done as described above.

### Immunohistochemistry

Patient tumor and normal tissues frozen in Allprotect tissue reagent were cut out and thawed. Tissues were soaked in 4% paraformaldehyde solution (Thomas Baker, 81847) for 24 h, put in cytomatrix (ThermoScientific, 6769006), and frozen at −80 °C. Frozen tissue samples were then subjected to cryo-sectioning using cryotome to get 6–8 µm thin sections. The tissue sections were stained with CERS2 (1:400 dilution) (SantaCruz, sc-390745) or Ceramide antibody (1:10 dilution) (Sigma, C8104) using the protocol previously described^69^. Confocal imaging of the samples was performed with Leica TCS SP8. The sections were visualized at ×40 oil immersion using LAS AF software. Z-stacking was performed, and images were acquired for each section. The images were processed using LAS X software. Image Quantification data were analyzed using two-tailed unpaired Student’s *t*-test.

### LC-MS/MS method and data analysis

Cell pellets were washed in ice-cold PBS, harvested, and centrifuged. The cell pellets were resuspended in PBS (200 µL), and homogenised by sonication. An aliquot (20 µL) was taken for protein estimation by BCA protein estimation kit. One milligram protein equivalent of each sample was used for lipid isolation using protocol described previously^69^. Only organic phase (OP) samples were extracted for quantitation of ceramides^69^.

OP sphingolipids were resuspended and separated using solvent A (99:1, water/formic acid) and solvent B (99:1, methanol/formic acid), both with 5 mM ammonium formate. The resulting OP extracts for each sample were sonicated for 15 sec, vortexed, and centrifuged at 13,000 rpm for 5 min. The clear supernatant was transferred to the autoinjector vial for LC-MS/MS analysis. Ceramides were analyzed using high-pressure UHPLC liquid chromatography (ExionLC AC, SCIEX, USA) coupled to a hybrid triple quadrupole/linear ion trap mass spectrometer (4500 QTRAP, SCIEX, USA). A Kinetex^®^ C8, 2.1 × 50 mm column (Phenomenex®) with a particle size of 1.7 μm was used, and oven temperature was kept at 60 °C. Total run time optimized was 10 min. Separation method used for ceramides was, Buffer A and B (20:80) for 0–1.2 min, 100% buffer B for 1.2–6.5 min, buffer A and B (20:80) for 6.5–10 min.

Ceramides were monitored using scheduled multiple reaction monitoring (MRM) with preidentified retention time. An MRM to enhanced product ion (EPI) scan was used. All parameters used were as described previously^69^. A standard curve was generated for absolute quantitation of ceramides. Ceramides in each sample were quantified using the same protocol as described previously^69^. Five technical replicates were run for each independent biological replicate (*n* = 4). Data were analyzed by ANOVA test.

### CERS2 in vitro activity assay

#### Preparation of membrane fractions

HEK 293T cells were transfected with CERS2 OE (AS1) and CERS2 OE (PC) plasmids as mentioned above. After 36 h, the membrane fraction of HEK 293T cells was prepared^48^. Cells were suspended in buffer A [50 mM HEPES-NaOH, pH 7.4, 150 mM NaCl, 10% glycerol, 1 mM DTT, 1 mM PMSF, and a 1X complete protease inhibitor cocktail], and lysed by sonication (30 amp, three cycle of 20 s on and 30 s off cycle). Homogenate (20 μL) were taken for protein estimation. Equal quantity of all samples (1.0 mg of protein equivalent) were processed. Cell debris were removed by centrifugation at 300 × *g*, 4 °C for 5 min. The supernatant was centrifuged at 100,000 × *g* (Optima XPN-100 Ultracentrifuge, Beckman Coulter, USA) at 4 °C for 30 min. The pellets (total membrane fraction) were suspended in buffer A.

#### In vitro CERS2 assay

Membrane fractions were incubated with 5 μM deuterium-labeled sphingosine (sphingosine-d7) (Sigma, 860657P) and 25 μM acyl-CoA of different acyl chain lengths C16:0 (palmitoyl)-CoA (Sigma, 870716P), C24:0 (lignoceroyl)-CoA (Sigma, 870724P), and C24:1 (nervonoyl)-CoA (Sigma, 870725P) in 100 μL of reaction buffer (buffer A containing 2 mM MgCl_2_ and 0.1% digitonin) at 37 °C for 30 min. The reaction was terminated by the addition of 375 μL of chloroform/methanol (1:2, v/v) and 1.25 μL of 12 M formic acid, followed by vigorous shaking. The mixture was subjected to phase separation by adding 125 μL of chloroform and 125 μL of H_2_O, followed by centrifugation (9000 × *g*, room temperature, 3 min). Lipids were recovered from the organic phase, dried, and suspended in chloroform/methanol (1:2, v/v). Deuterium-labeled ceramide (ceramide-d7) species were detected by LC/MS-MS.

#### Estimation of Ceramide-d7 using LC-MS/MS

LC-MS/MS was performed using the same conditions as mentioned above. Ceramides were monitored using Scheduled MRM. All parameters are described in Supplementary Table 5. Data analysis and quantification were performed using MultiQuant software (version 3.0.2; SCIEX, USA). Six technical replicates were run for each independent biological replicate (*n* = 4). Data were analyzed using ANOVA test.

### Proliferation assay

BT-474 cells (~2.5 × 10^5^/well) were seeded in a six-well plate as mentioned above. After 24 h of incubation, cells were transfected (according to above mentioned procedure) with control vector, CERS2 OE (PC), and CERS2 OE (AS1) plasmids, and incubated for 24 h. Transfected BT-474 cells (5000 cells/well) were then seeded in a 96 well plate for 24, 48, and 72 h in complete DMEM media, and incubated at 37 °C. Cell proliferation was quantified using MTT assay^70^. Absorbance reading was taken at 590 nm in iMark^TM^ Microplate Absorbance Bio-RAD plate reader at different time points (24, 48, and 72 h). The experiment was performed five independent times, and data were analyzed by two-way ANOVA.

### Scratch wound migration assay^71^

BT-474 cells (~2.5 × 10^5^/well) were seeded in a six-well plate as mentioned above. After 24 h of incubation, cells were transfected (as mentioned above) with control vector, CERS2 OE (PC), and CERS2 OE (AS1) plasmids, and incubated further at 37 °C for 24 h. As cells attain around 90% confluency, the media was aspirated, and a scratch was made by sterile 200 μL pipette tip. Boundaries on each side of the scratch were demarcated by a borderline as shown in Fig. 7C. The wells were washed gently with 1X DPBS twice to get rid of the scraped floating cells, and complete DMEM media was added to the wells. The scratch was imaged immediately for the zero-time point at ×10 magnification in bright field using an inverted microscope (Nikon Inverted LED microscope, Eclipse Ts2, Minato, Tokyo, Japan), and cells were further incubated under similar conditions. The scratch was monitored, imaged, and documented at 6, 18, 24, and 36 h at the same position. A mark was made on the plastic plate as a reference point to ensure that the same area was imaged every time over the course of the experiment. The number of cells migrating into the scratch wound was monitored, and counted using Capture 2.0 image software. The experiment was performed five times independently, and data were analyzed by two-way ANOVA.

### Annexin V-PI apoptosis assay

BT-474 cells (1 × 10^5^) were seeded in 24-well plate and incubated for 24 h. Cells were transfected with control vector or CERS2 OE (PC) or CERS2 OE (AS1) plasmids in respective wells as mentioned above. After 48 h, cells were trypsinized and washed with 1X PBS followed by a second wash with 1X binding buffer. Cells were further resuspended into 100 µL of 1X binding buffer. Mixture of Annexin V-FITC (5 µL) and propidium iodide (PI) (5 µL) (BD Biosciences, 556547) were added followed by incubation at room temperature for 15 min in the dark. The stained cells were acquired in BD FACSverse with appropriate gating.

### Statistical analyses

All statistical analyses were performed using GraphPad Prism version 7.0 (GraphPad Software, La Jolla, CA, USA). Data were analyzed by ANOVA or two-tailed unpaired Student’s *t*-test or two-way ANOVA.

## Supplementary information

Supplementary Informations

Supplementary Figure 1

Supplementary Figure 2

Supplementary Figure 3

Supplementary Figure 4

Supplementary Figure 5

Supplementary Figure 6

Supplementary Figure 7

Supplementary Figure 8

Supplementary Figure 9

Supplementary Figure 10

Supplementary Figure 11

Supplementary Figure 12

Supplementary Figure 13

Supplementary Figure 14

Supplementary Figure 15

Supplementary Figure 16

Supplementary Figure 17

Supplementary Figure 18

Supplementary Figure 19

Supplementary Figure 20

Data Set 1

Data Set 2

Data Set 3

Data Set 4

Data Set 5

Data Set 6

Data Set 7

Data Set 8

Data Set 9

Data Set 10

Data Set 11

## References

[1] Hannun YA, Obeid LM (2008). Principles of bioactive lipid signalling: lessons from sphingolipids. Nat. Rev. Mol. Cell Biol..

[2] Hannun YA, Obeid LM (2018). Sphingolipids and their metabolism in physiology and disease. Nat. Rev. Mol. Cell Biol..

[3] Futerman AH, Hannun YA (2004). The complex life of simple sphingolipids. EMBO Rep..

[4] Ponnusamy S (2010). Sphingolipids and cancer: ceramide and sphingosine-1-phosphate in the regulation of cell death and drug resistance. Future Oncol..

[5] Aoyagi T, Nagahashi M, Yamada A, Takabe K (2012). The role of sphingosine-1-phosphate in breast cancer tumor-induced lymphangiogenesis. Lymphat Res Biol..

[6] Ogretmen B (2018). Sphingolipid metabolism in cancer signalling and therapy. Nat. Rev. Cancer.

[7] Ryland LK, Fox TE, Liu X, Loughran TP, Kester M (2011). Dysregulation of sphingolipid metabolism in cancer. Cancer Biol. Ther..

[8] Saddoughi SA, Song P, Ogretmen B (2008). Roles of bioactive sphingolipids in cancer biology and therapeutics. Subcell. Biochem.

[9] Hannun YA, Obeid LM (2002). The ceramide-centric universe of lipid-mediated cell regulation: stress encounters of the lipid kind. J. Biol. Chem..

[10] Moro K (2018). Ceramide species are elevated in human breast cancer and are associated with less aggressiveness. Oncotarget.

[11] Schiffmann S (2009). Ceramide synthases and ceramide levels are increased in breast cancer tissue. Carcinogenesis.

[12] Levy M, Futerman AH (2010). Mammalian ceramide synthases. IUBMB Life.

[13] Wegner MS, Schiffmann S, Parnham MJ, Geisslinger G, Grosch S (2016). The enigma of ceramide synthase regulation in mammalian cells. Prog. Lipid Res..

[14] Erez-Roman R, Pienik R, Futerman AH (2010). Increased ceramide synthase 2 and 6 mRNA levels in breast cancer tissues and correlation with sphingosine kinase expression. Biochem. Biophys. Res. Commun..

[15] Mullen TD, Hannun YA, Obeid LM (2012). Ceramide synthases at the centre of sphingolipid metabolism and biology. Biochem. J..

[16] Wang H (2012). Expression and prognostic significance of a new tumor metastasis suppressor gene LASS2 in human bladder carcinoma. Med. Oncol..

[17] Ke RH, Wang Y, Mao Y, Zhang J, Xiong J (2014). Decreased expression of LASS2 is associated with worse prognosis in meningiomas. J. Neurooncol..

[18] Zhang Q (2019). Clinical and pathological significance of Homo sapiens ceramide synthase 2 (CerS-2) in diverse human cancers. Biosci. Rep..

[19] Gong M (2012). KLF6/Sp1 initiates transcription of the TMSG-1 gene in human prostate carcinoma cells: an exon involved mechanism. J. Cell Biochem..

[20] Moriya Y (2012). Tumor suppressive microRNA-133a regulates novel molecular networks in lung squamous cell carcinoma. J. Hum. Genet..

[21] Yu B (2012). miR-221 and miR-222 promote Schwann cell proliferation and migration by targeting LASS2 after sciatic nerve injury. J. Cell Sci..

[22] Ma C (2002). Identification of tumor metastasis-related gene TMSG-1 by mRNA differential display. Sci. China C. Life Sci..

[23] Di C (2019). Function, clinical application, and strategies of pre-mRNA splicing in cancer. Cell Death Differ..

[24] Escobar-Hoyos L, Knorr K, Abdel-Wahab O (2019). Aberrant RNA splicing in cancer. Annu Rev. Cancer Biol..

[25] Chalfant CE (2002). De novo ceramide regulates the alternative splicing of caspase 9 and Bcl-x in A549 lung adenocarcinoma cells. Dependence on protein phosphatase-1. J. Biol. Chem..

[26] Shkreta L (2008). Anticancer drugs affect the alternative splicing of Bcl-x and other human apoptotic genes. Mol. Cancer Ther..

[27] Pal S (2019). A localized chimeric hydrogel therapy combats tumor progression through alteration of sphingolipid metabolism. ACS Cent. Sci..

[28] Stricker TP (2017). Robust stratification of breast cancer subtypes using differential patterns of transcript isoform expression. PLoS Genet..

[29] Eswaran J (2012). Transcriptomic landscape of breast cancers through mRNA sequencing. Sci. Rep..

[30] Zhao W, Hoadley KA, Parker JS, Perou CM (2016). Identification of mRNA isoform switching in breast cancer. BMC Genomics.

[31] Ciriello G (2015). Comprehensive molecular portraits of invasive lobular breast cancer. Cell.

[32] Tabrez SS, Sharma RD, Jain V, Siddiqui A, Mukhopadhyay A (2017). Differential alternative splicing coupled to nonsense-mediated decay of mRNA ensures dietary restriction-induced longevity. Nat. Commun..

[33] Bastien RRL (2012). PAM50 breast cancer subtyping by RT-qPCR and concordance with standard clinical molecular markers. BMC Med. Genomics.

[34] David CJ, Manley JL (2010). Alternative pre-mRNA splicing regulation in cancer: pathways and programs unhinged. Genes Dev..

[35] Li M, Liu Y (2016). Topoisomerase I in human disease pathogenesis and treatments. Genomics Proteom. Bioinforma..

[36] Carr HS, Zuo Y, Oh W, Frost JA (2013). Regulation of focal adhesion kinase activation, breast cancer cell motility, and amoeboid invasion by the RhoA guanine nucleotide exchange factor Net1. Mol. Cell Biol..

[37] Kurosaki T, Maquat LE (2016). Nonsense-mediated mRNA decay in humans at a glance. J. Cell Sci..

[38] Vandenbroucke II, Vandesompele J, Paepe AD, Messiaen L (2001). Quantification of splice variants using real-time PCR. Nucleic Acids Res..

[39] Virtue S, Dale M, Sethi JK, Vidal-Puig A (2010). LEM-PCR: a method for determining relative transcript isoform proportions using real-time PCR without a standard curve. Genome.

[40] Hoang TX, Duong CN, Kim JY (2017). Identification and characterization of a splicing variant in the 5’ UTR of the human TLR5 gene. BioMed. Res. Int..

[41] Venkataraman K, Futerman AH (2002). Do longevity assurance genes containing Hox domains regulate cell development via ceramide synthesis?. FEBS Lett..

[42] Tidhar R (2012). Acyl chain specificity of ceramide synthases is determined within a region of 150 residues in the Tram-Lag-CLN8 (TLC) domain. J. Biol. Chem..

[43] Spassieva S (2006). Necessary role for the Lag1p motif in (Dihydro)ceramide synthase activity. J. Biol. Chem..

[44] Takeda T (2019). Upregulation of IGF2R evades lysosomal dysfunction-induced apoptosis of cervical cancer cells via transport of cathepsins. Cell Death Dis..

[45] Shen S, Wang Y, Wang C, Wu YN, Xing Y (2016). SURVIV for survival analysis of mRNA isoform variation. Nat. Commun..

[46] Harrell FE, Califf RM, Pryor DB, Lee KL, Rosati RA (1982). Evaluating the yield of medical tests. JAMA.

[47] Laviad EL, Kelly S, Merrill AH, Futerman AH (2012). Modulation of ceramide synthase activity via dimerization. J. Biol. Chem..

[48] Sassa T, Hirayama T, Kihara A (2016). Enzyme activities of the ceramide synthases CERS2-6 are regulated by phosphorylation in the C-terminal region. J. Biol. Chem..

[49] Xu X (2014). Silencing of LASS2/TMSG1 enhances invasion and metastasis capacity of prostate cancer cell. J. Cell Biochem..

[50] Mei F (2015). LASS2/TMSG1 inhibits growth and invasion of breast cancer cell in vitro through regulation of vacuolar ATPase activity. Tumour Biol..

[51] Zou P (2018). Silencing of vacuolar ATPase c subunit ATP6V0C inhibits the invasion of prostate cancer cells through a LASS2/TMSG1-independent manner. Oncol. Rep..

[52] Wang H (2017). LASS2 inhibits growth and invasion of bladder cancer by regulating ATPase activity. Oncol. Lett..

[53] Pagliarini V, Naro C, Sette C (2015). Splicing regulation: a molecular device to enhance cancer cell adaptation. Biomed. Res. Int..

[54] Kang H, Kim H, Lee S, Youn H, Youn B (2019). Role of metabolic reprogramming in epithelial-mesenchymal transition (EMT). Int. J. Mol. Sci..

[55] Tidhar R (2018). Eleven residues determine the acyl chain specificity of ceramide synthases. J. Biol. Chem..

[56] Espaillat M, Shamseddine A, Adada M, Hannun Y, Obeid L (2015). Ceramide and sphingosine-1-phosphate in cancer, two faces of the sphinx. Transl. Cancer Res..

[57] Garcia-Gonzalez V (2018). Ceramide metabolism balance, a multifaceted factor in critical steps of breast cancer development. Int. J. Mol. Sci..

[58] Hartmann D (2012). Long chain ceramides and very long chain ceramides have opposite effects on human breast and colon cancer cell growth. Int. J. Biochem. Cell Biol..

[59] Edmond V (2015). Downregulation of ceramide synthase-6 during epithelial-to-mesenchymal transition reduces plasma membrane fluidity and cancer cell motility. Oncogene.

[60] Chen Y (2017). The role of LASS2 in regulating bladder cancer cell tumorigenicity in a nude mouse model. Onco. Lett..

[61] Fan S (2015). CERS2 suppresses tumor cell invasion and is associated with decreased V-ATPase and MMP-2/MMP-9 activities in breast cancer. J. Cell Biochem..

[62] Tran B, Bedard PL (2011). Luminal-B breast cancer and novel therapeutic targets. Breast Cancer Res..

[63] Mane A (2015). A comparison of clinical features, pathology and outcomes in various subtypes of breast cancer in Indian women. J. Clin. Diagn. Res..

[64] Cheang MC (2009). Ki67 index, HER2 status, and prognosis of patients with luminal B breast cancer. J. Natl Cancer Inst..

[65] Li ZH, Hu PH, Tu JH, Yu NS (2016). Luminal B breast cancer: patterns of recurrence and clinical outcome. Oncotarget.

[66] Cox DR (1972). Regression models and life-tables. J. R. Stat. Soc. Ser. B (Methodol.).

[67] Cox DR (1975). Partial likelihood. Biometrika.

[68] Johnson, N. L., Kotz, S. & Balakrishnan, N. *Continuous Univariate Distributions* 2nd edn, Vol. 2 (Wiley, 1995).

[69] Medatwal, N. & Dasgupta, U. *Analysis of Membrane Lipids* (eds Prasad, R. & Singh, A.) 103–117 (Springer Protocols Handbooks, Springer, 2020).

[70] Li T (2015). Umbilical cord-derived mesenchymal stem cells promote proliferation and migration in MCF-7 and MDA-MB-231 breast cancer cells through activation of the ERK pathway. Oncol. Rep..

[71] Saini M, Verma A, Mathew SJ (2018). SPRY2 is a novel MET interactor that regulates metastatic potential and differentiation in rhabdomyosarcoma. Cell Death Dis..

