# Supplementary material for: Alternative splicing of ceramide synthase 2 alters levels of specific ceramides and modulates cancer cell proliferation and migration in Luminal B breast cancer subtype
Source: Cell Death Dis. 2021 Feb 10;12(2):171. doi: 10.1038/s41419-021-03436-x (PMC7876150; doi:10.1038/s41419-021-03436-x)
Supplement: Supplementary file 1 — Supplementary Informations [file 41419_2021_3436_MOESM1_ESM.docx]

**Supplementary Informations**

| **Supplementary Table 1.** (A) List of primers (human) used for validation of endogenous gene expression by Real-Time PCR. (B) List of Primers (human) used for validation of Alternative Splicing events.  A | | |
| --- | --- | --- |
| **Gene Name** | **Forward primer (5' to 3')** | **Reverse primer (5' to 3')** |
| *CerS1* | ACGCTACGCTATACATGGACAC | AGGAGGAGACGATGAGGATGAG |
| *CerS2* | GGTCCCTGCTCTTCAGCATTG | GGATGACCAGTCGGGTGATG |
| *CerS3* | ACATTCCACAAGGCAACCATTG | CTCTTGATTCCGCCGACTCC |
| *CerS4* | CTTCGTGGCGGTCATCCTG | TGTAACAGCAGCACCAGAGAG |
| *CerS5* | TGTAACAGCAGCACCAGAGAG | GCCAGCACTGTCGGATGTC |
| *CerS6* | GGGATCTTAGCCTGGTTCTGG | GCCTCCTCCGTGTTCTTCAG |
| *β-actin* | ATTGGCAATGAGCGGTTCC | GGTAGTTTCGTGGATGCCACA |

| B | | |
| --- | --- | --- |
| **Gene/ Oligo Name** | **Sequence (5'-3')** | **Position** |
| *TOP1_F1* | GAACAAGCAGCCCGAGGATG | Exon-16 |
| *TOP1_R1* | GCTGCTGTAGCGTGATGGAG | Exon-17 |
| *TOP1_F15-17* | CGAACTGGTATTCTGAATAAGC | Exon-15-17 junction |
| *RHOA_F1* | CCAGTTCCCAGAGGTGTATG | Exon-2 |
| *RHOA_R1* | GGGCTGTCGATGGAAAAACAC | Exon-3 |
| *RHOA_F2-4* | GGAAAGCAGAAAACATCCCAG | Exon-2-4 junction |
| *RHOA_R2* | CGTTGGGACAGAAATGCTTGAC | Exon-4 |
| *CERS2_F3* | GTCAAGCGAAAGGATTTCAAGG | Exon-7-8 junction |
| *CERS2_R3* | GGATGACCAGTCGGGTGATG | Exon-9 |
| *CERS2_E7-9F* | GTCAAGCGAAAGTCAGCCAAG | Exon-7-9 junction |

**Supplementary Table 2.** Table showing list of b and y ions for unique junction-spanning peptides (Exon7-8) for full-length PC protein and (Exon7-9) alternatively spliced AS1 coding protein included in the Multiple Reaction Monitoring method used, and parameters for estimation of daughter ions.

| **Peptide Sequence** | **Theoretical m/z** | **Product ion (Q1)** | **Product ion (Q3)** | | | | **Collision Energy (V)** |
| --- | --- | --- | --- | --- | --- | --- | --- |
|  |  |  | **b** | **Status*** | **y** | **Status*** |  |
| SIASDVKRKDF (Full-length) | 633.3461 | 633.3461 | 88.0393 | ✗ | 1265.68483 | ✗ | 36 |
|  |  |  | 201.12337 | ✓ | 1178.6528 | ✗ |  |
|  |  |  | 272.16048 | ✓ | 1065.56874 | ✗ |  |
|  |  |  | 359.19251 | ✓ | 994.53162 | ✓ |  |
|  |  |  | 474.21945 | ✓ | 907.49959 | ✓ |  |
|  |  |  | 573.28787 | ✓ | 792.47265 | ✓ |  |
|  |  |  | 701.38283 | ✓ | 693.40424 | ✓ |  |
|  |  |  | 857.48394 | ✓ | 565.30927 | ✓ |  |
|  |  |  | 985.57891 | ✓ | 409.20816 | ✓ |  |
|  |  |  | 1100.60585 | ✓ | 281.1132 | ✓ |  |
|  |  |  | 1247.67426 | ✗ | 166.08626 | ✓ |  |
|  |  |  |  |  |  |  |  |
| SIASDVKRKSAKMF (AS1) | 784.4349 | 784.4349 | 88.0393 | ✗ | 1567.86248 | ✗ | 46 |
|  |  |  | 201.12337 | ✓ | 1480.83045 | ✗ |  |
|  |  |  | 272.16048 | ✓ | 1367.74638 | ✓ |  |
|  |  |  | 359.19251 | ✓ | 1296.70927 | ✓ |  |
|  |  |  | 474.21945 | ✓ | 1209.67724 | ✓ |  |
|  |  |  | 573.28787 | ✓ | 1094.6503 | ✓ |  |
|  |  |  | 701.38283 | ✓ | 995.58188 | ✗ |  |
|  |  |  | 857.48394 | ✗ | 867.48692 | ✗ |  |
|  |  |  | 985.57891 | ✗ | 711.38581 | ✓ |  |
|  |  |  | 1072.61093 | ✗ | 583.29085 | ✓ |  |
|  |  |  | 1143.64805 | ✗ | 496.25882 | ✓ |  |
|  |  |  | 1271.74301 | ✗ | 425.2217 | ✓ |  |
|  |  |  | 1402.7835 | ✗ | 297.12674 | ✓ |  |
|  |  |  | 1549.85191 | ✗ | 166.08626 | ✓ |  |
| * All fragment (daughter) ions with signal to noise more than 3 are identified and marked by ✓, and those not identified marked by ✗. | | | | | | | |

| Supplementary Table 3. Table showing the clinical and pathological information of Luminal B breast cancer female patients of Indian origin included in this study. | | | | | | | | |  |
| --- | --- | --- | --- | --- | --- | --- | --- | --- | --- |
| S. No. | Age^a^ | Family History^b^ | Menopausal Status^c^ | Lymph Vascular Invasion^d^ | Clinical Stage^e^ | Pathological Stage^f^ | Grade^g^ | | |
| P1 | 33/F | N | Pre | - | T_4b_N_0_M_0_ | yT_0_N_0_M_0_ | N/A | | |
| P2 | 79/F | N | Post | - | T_2_N_0_M_0_ | T_2_N_0_ | 3 | | |
| P3 | 72/F | N | Post | + | T_3_N_0_M_0_ | T_2_N_1A_ | 3 | | |
| P4 | 68/F | N | Post | + | T_2_N_1_M_0_ | yT_2_N_3a_ | 3 | | |
|  |  |  |  |  |  |  |  | | |
|  |  |  |  |  |  |  |  | | |
| P5 | 36/F | N | Pre | + | T_3_N_0_M_0_ | yT_3_N_2a_ | 3 | | |
| P6 | 30/F | N | Pre | - | T_2_N_0_M_0_ | yT_2_N_0_ | N/A | | |
| P7 | 51/F | N | Post | + | T_2_N_0_M_0_ | yT_2_N_1a_ | 3 | | |
| P8 | 49/F | N | Post | + | T_2_N_1_M_0_ | T_2_N_0_ | 2 | | |
| P9 | 53/F | Y | Post | - | T_2_N_0_M_0_ | T_2_N_0_ | 2 | | |
| P10 | 37/F | Y | Pre | + | T_2_N_1_M_0_ | yT_2_N_1a_ | 3 | | |
| P11 | 35/F | N | Pre | + | T_2_N_0_M_0_ | yT_2_N_1a_ | 3 | | |
| P12 | 54/F | N | Post | + | T_2_N_1_M_0_ | yT_2_N_3a_ | 3 | | |
| P13 | 47/F | Y | Pre | + | T_2_N_1_M_0_ | T_2_N_1a_ | 3 | | |
| P14 | 40/F | N | Pre | - | T_4b_N_2a_M_0_ | yT_0_N_0_M_0_ | N/A | | |
| P15 | 57/F | N | Post | - | T_3_N_0_M_0_ | T_2_N_0_M_0_ | 2 | | |
| P16 | 55/F | N | Post | + | T_4b_ N_2_M_1_ | yT_1mi_ N_2_M_1_ | 2 | | |
| P17 | 55/F | N | Post | - | T_2_N_0_M_0_ | T_4b_N_2a_M_0_ | 2 | | |
| P18 | 74/F | N | Post | + | T_3_N_1_M_0_ | T_2_N_2a_M_0_ | 2 | | |
| P19 | 78/F | N | Post | - | T_4b_N_1_M_0_ | T_2_N_2_M_0_ | 2 | | |
| P20 | 42/F | N | Pre | - | T_4b_N_0_M_0_ | yT_0_N_0_M_0_ | N/A | | |
| P21 | 36/F | N | Pre | - | T_2_N_1_M_1_ | yT_1_N_0_M_0_ | N/A | | |
| P22 | 64/F | N | Post | - | T_4b_N_1_M_0_ | T_4b_N_2_M_0_ | 2 | | |
| a: Age (Years); b: History of patients regarding cancer occurrence in family. c: Menopausal status of patient when cancer was diagnosed. d: Extent of lymph channels or blood vessels invaded by breast cancer cells as detected in the patient. e: Tumor staging done by clinician according to parameters described by American Joint Committee for Cancer Control (AJCC, 8^th^ edition, 2018) including Tumor size (T), number of lymph nodes invaded (N) and extent of metastasis (M). f: Tumor staging done by pathologist after surgery/biopsy from tumor tissue for tumor size (T), number of lymph nodes invaded (N), extent of metastasis (M) and staging after chemotherapy (Y). g: Grade signifies the aggressiveness of cancer on the basis of tumor description as per pathology report after biopsy. | | | | | | | |  |  |

| **Supplementary Table 4.** Table showing junction information of Exon 8 (CERS2), used for Survival analysis of Luminal B patients of TCGA-BRCA cohort. | | | | |
| --- | --- | --- | --- | --- |
| **Term** | **Start** | **Stop** | **Strand** | **Association** |
| EX419712 | 150967074 | 150967202 | - | Exon 8 |
| JUNC51126 | 150966862 | 150967073 | - | Flanking Junction |
| JUNC51127 | 150966862 | 150967391 | - | Skipping Junction |
| JUNC51128 | 150966862 | 150967663 | - | Skipping Junction |
| JUNC51134 | 150967202 | 150967391 | - | Flanking Junction |
| JUNC51135 | 150967202 | 150967663 | - | Flanking Junction |

| **Supplementary Table 5.** Table showing parameters for estimation of ceramide and deuterium-labelled ceramide species for *in vitro* Ceramide synthase 2 (CERS2) activity assay. | | | |
| --- | --- | --- | --- |
| **Ceramide Species** | **Precursor ion (Q1)** | **Product ion (Q3)** | **Collision Energy (V)** |
| d18:1/C16:0 Cer | 538.5 | 264.2 | 35 |
| d18:1/C16:0 Cer-d7 | 545.2 | 271.2 | 35 |
| d18:1/C24:1 Cer | 648.9 | 264.2 | 41 |
| d18:1/C24:1 Cer-d7 | 655.3 | 271.2 | 41 |
| d18:1/C24:0 Cer | 650.6 | 264.2 | 41 |
| d18:1/C24:0 Cer-d7 | 657.3 | 271.2 | 41 |
| Ceramide (Cer)-d7: deuterium-labeled ceramide | | |  |

**Supplementary Figure Legends**

**Supplementary Figure 1.** (**A, B**) KEGG pathways, Gene Ontology (GO)-Biological Processes, and Protein domains of alternatively spliced genes predicted by DAVID Functional Annotation tool for Luminal A subtype using RNA sequencing data from TCGA-BRCA cohort. The number adjacent to each bar represents number of events identified in that category. The listed pathways and GO terms have a *p* value ≤ 0.05 Fisher Exact Test and FDR ≤ 15.

**Supplementary Figure 2.** (**A, B**) KEGG pathways, Gene Ontology (GO)-Biological Processes, and Protein domains of alternatively spliced genes predicted by DAVID Functional Annotation tool for Luminal B subtype using RNA sequencing data from TCGA-BRCA cohort. The number adjacent to each bar represents number of events identified in that category. The listed pathways and GO terms have a *p* value ≤ 0.05 Fisher Exact Test and FDR ≤ 15.

**Supplementary Figure 3.** (**A, B**) KEGG pathways, Gene Ontology (GO)-Biological Processes, and Protein domains of alternatively spliced genes predicted by DAVID Functional Annotation tool for Basal subtype using RNA sequencing data from TCGA-BRCA cohort. The number adjacent to each bar represents number of events identified in that category. The listed pathways and GO terms have a *p* value ≤ 0.05 Fisher Exact Test and FDR ≤ 15.

**Supplementary Figure 4.** (**A, B**) KEGG pathways, Gene Ontology (GO)-Biological Processes, and Protein domains of alternatively spliced genes predicted by DAVID Functional Annotation tool for HER2+ subtype using RNA sequencing data from TCGA-BRCA cohort. The number adjacent to each bar represents number of events identified in that category. The listed pathways and GO terms have a *p* value ≤ 0.05 Fisher Exact Test and FDR ≤ 15.

**Supplementary Figure 5.** Diagrammatic representation of alternative splicing AS1 event from CERS2 pre mRNA.

**Supplementary Figure 6.** Alignment of nucleotide sequence of CERS2 full-length protein coding transcript and alternatively spliced AS1 transcript.

**Supplementary Figure 7.** Amino acid sequence alignment of full length CERS2 protein (380 amino acids) encoded by protein coding (PC) transcript and protein (337 amino acids) encoded by alternatively spliced AS1 transcript.

**Supplementary Figure 8.** Diagrammatic representation of AS2 alternative splicing event from CERS2 pre-mRNA.

**Supplementary Figure 9.** Alignment of nucleotide sequence of CERS2 full-length protein coding transcript and alternatively spliced AS2 transcript. Red letters in lower case denotes 179 base pair intron between exon 6 and 7, of which the first 33 base pairs are retained in the AS2 transcript.

**Supplementary Figure 10.** Amino acid sequence alignment of full length CERS2 protein (380 amino acids) encoded by protein coding (PC) transcript and protein (174 amino acids) encoded by alternatively spliced AS2 transcript.

**Supplementary Figure 11.** Agarose gel electrophoresis of RT-PCR amplified products of *CERS2* using event specific primer. *CERS2* transcript amplified from BT-474, BT-474 (+Vector), CERS2 OE (PC) and CERS2 OE (AS1) cells using same primer pair (forward from exon 7 and reverse from exon 9) shows a band with 129 base pair reduction due to lack of exon 8 for CERS2 OE (AS1) cells.

**Supplementary Figure 12.** Schematic representation of full length CERS2 and AS1 protein showing the distinct domains encoded by them.

**Supplementary Figure 13.** Amino acid sequence alignment of full length CERS2 protein and AS1 protein showing three distinct domains of CERS2: Hox-like, TLC and Lag1p domains. Blue box represents the transmembrane domains. The yellow highlighted region indicates 12 residues important for its activity, and conserved histidine residues are marked red.

**Supplementary Figure 14.** Immunoblot from untransfected, Vector transfected, CERS2 OE(PC), and CERS2 OE (AS1) transfected HEK293T cell lysates using CERS2, and β-actin antibodies. Overexpressed protein coding (PC) (lane 3) and endogenous PC (lane 4) band are marked by blue asterix, and overexpressed AS1 (lane 4) by red asterix.

**Supplementary Figure 15.** (A) Coomassie stain of immunoprecipitated endogenous CERS2 protein from BT-474 cells showing the gel slice (red box) cut out for in gel chymotrypsin digestion. The black arrows show the three gel slices cut out from the gel. (B, C) Peak area representing abundance of few selected daughter ions as quantified from 633.346 Da m/z parent ion corresponding to PC/FL (B), and from 784.434 Da m/z corresponding to AS1/SF protein (C). (PC/FL= Protein coding/Full length protein; AS1/SF= Alternatively spliced 1/Spliced form).

**Supplementary Figure 16.** Representative immunofluorescence images show increased ceramide levels in tumor tissue sections as compared to normal tissue in Luminal B patients. Ceramide staining is represented by green, and nuclear staining is represented by blue color.

**Supplementary Figure 17.** Immunoblot of 4 pairs of tumors and adjoining normal patient tissue with CERS2 antibody after long exposure showing higher levels of PC and AS1 protein product in Luminal B tumors as compared to adjoining normal tissue. Protein corresponding to PC transcript is marked by blue asterix and to AS1 transcript by red asterix. β-actin is used as the loading control.

**Supplementary Figure 18.** Immunoblot of BT-474, BT-474 (+Vector), CERS2 OE (PC), and CERS2 OE (AS1) protein using CERS1, CERS5, and CERS6 antibodies do not show any significant alteration.

**Supplementary Figure 19.** (**A-C**) Comparative quantitation of Ceramide C16:0 (**A**), Ceramide C18:0 (**B**) and Ceramide C20:0 (**C**) in BT-474, BT-474 (+Vector), CERS2 OE (PC) and CERS2 OE (AS1) cells do not show a significant change.

**Supplementary Figure 20.** (**A, B**) Representative flow cytometry scans of BT-474, BT-474 (+Vector), CERS2 OE(PC), and CERS2 OE (AS1) cells using Annexin-FITC/Propidium Iodide staining. (**B**) Percentage of cell death (mean ± SD, n = 4) in BT-474, BT-474 (+Vector), CERS2 OE(PC), and CERS2 OE (AS1) cells after 48 h of transfection.
