# Supplementary figures and images for: Alternative splicing of ceramide synthase 2 alters levels of specific ceramides and modulates cancer cell proliferation and migration in Luminal B breast cancer subtype
Source: Cell Death Dis. 2021 Feb 10;12(2):171. doi: 10.1038/s41419-021-03436-x (PMC7876150; doi:10.1038/s41419-021-03436-x)

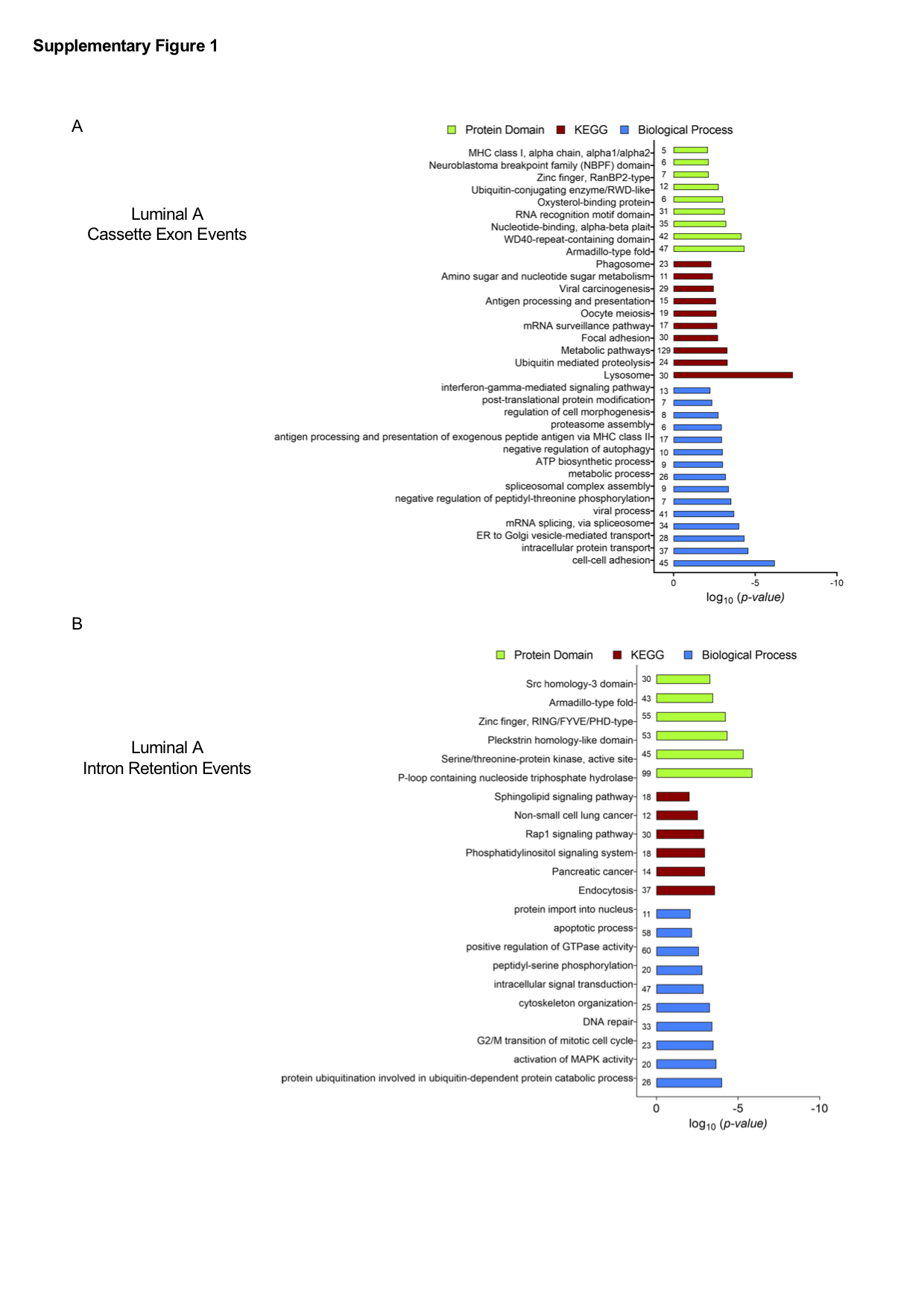

Supplement: Supplementary file 2 — Supplementary Figure 1 [file 41419_2021_3436_MOESM2_ESM.tif]

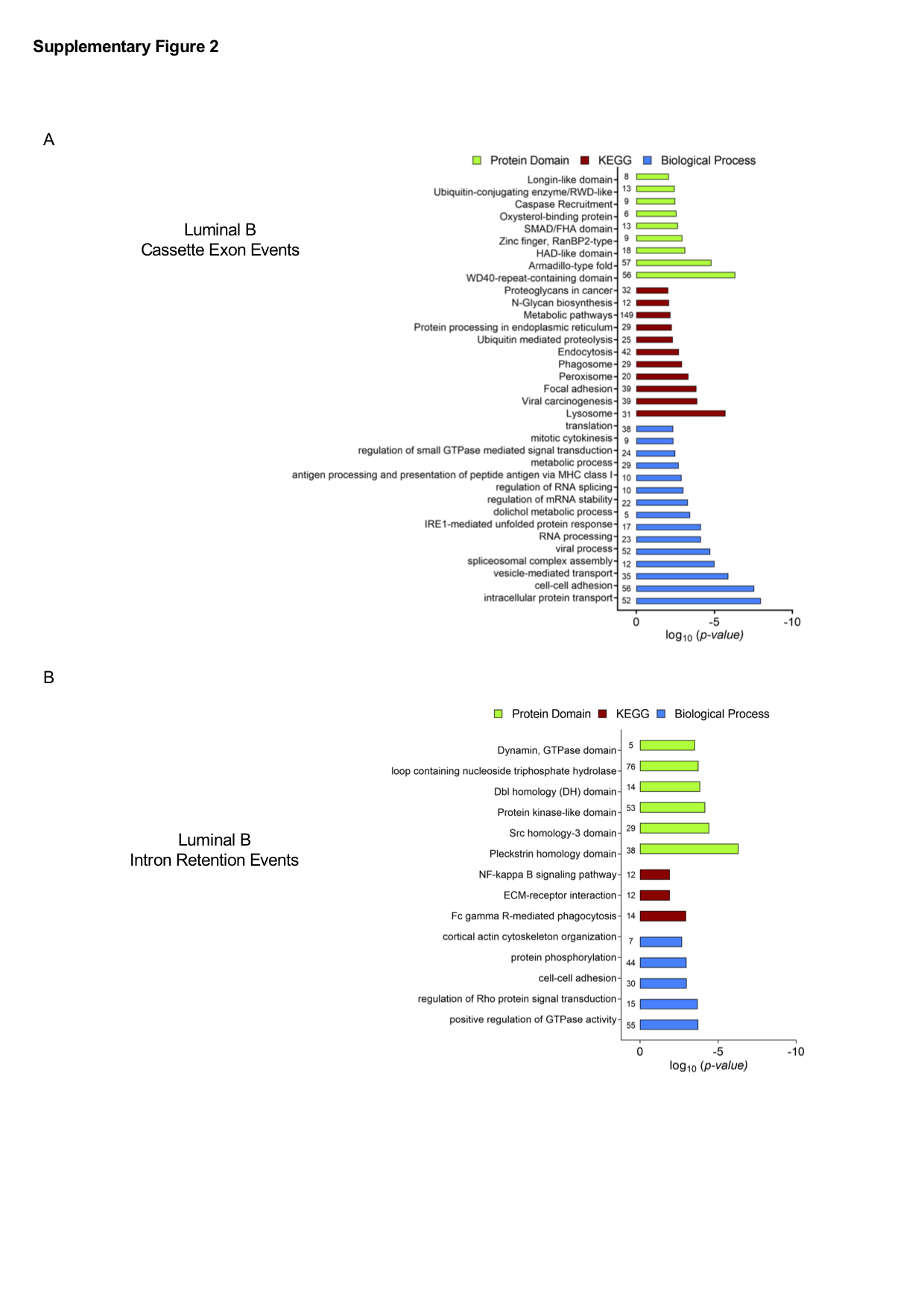

Supplement: Supplementary file 3 — Supplementary Figure 2 [file 41419_2021_3436_MOESM3_ESM.tif]

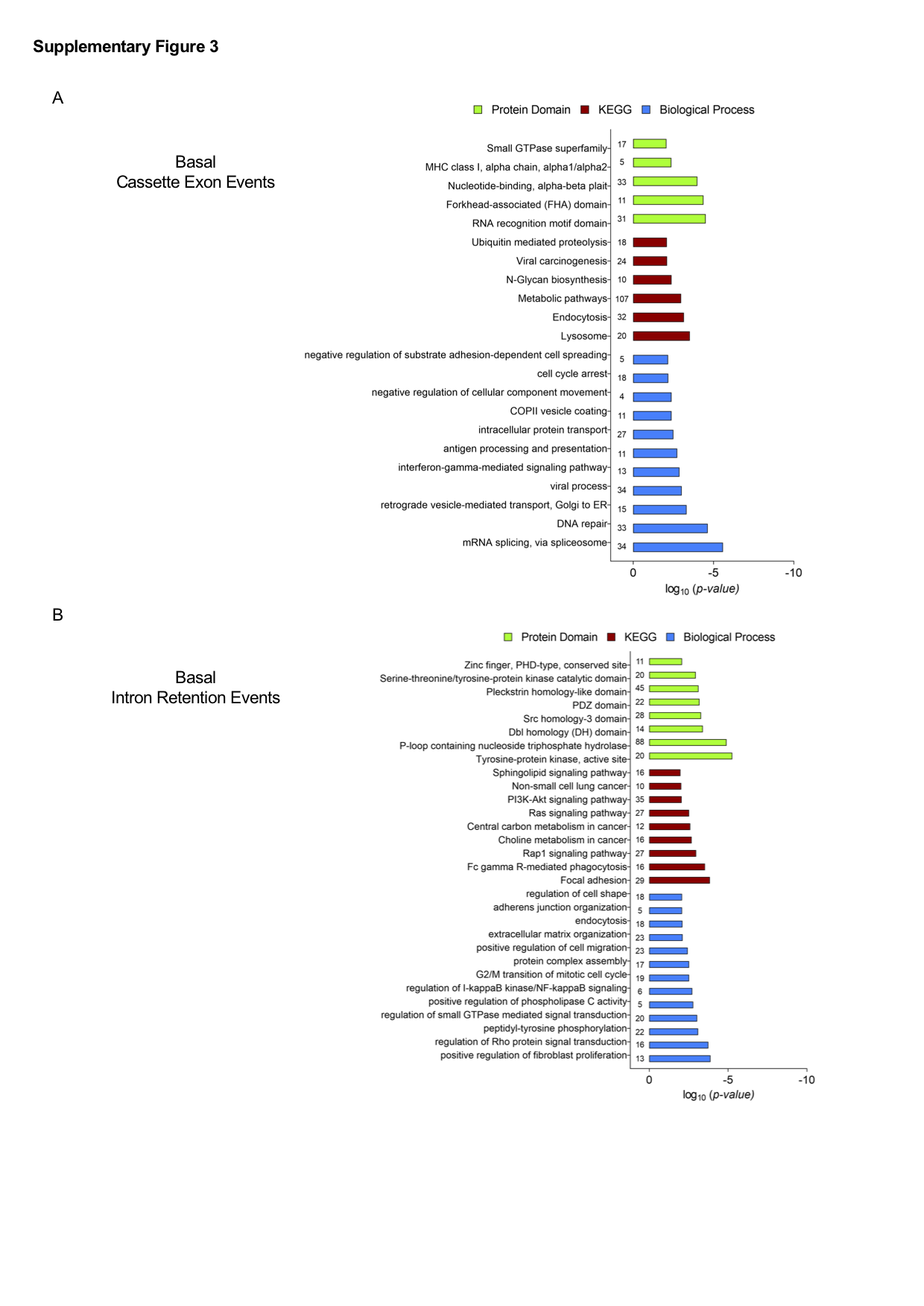

Supplement: Supplementary file 4 — Supplementary Figure 3 [file 41419_2021_3436_MOESM4_ESM.tif]

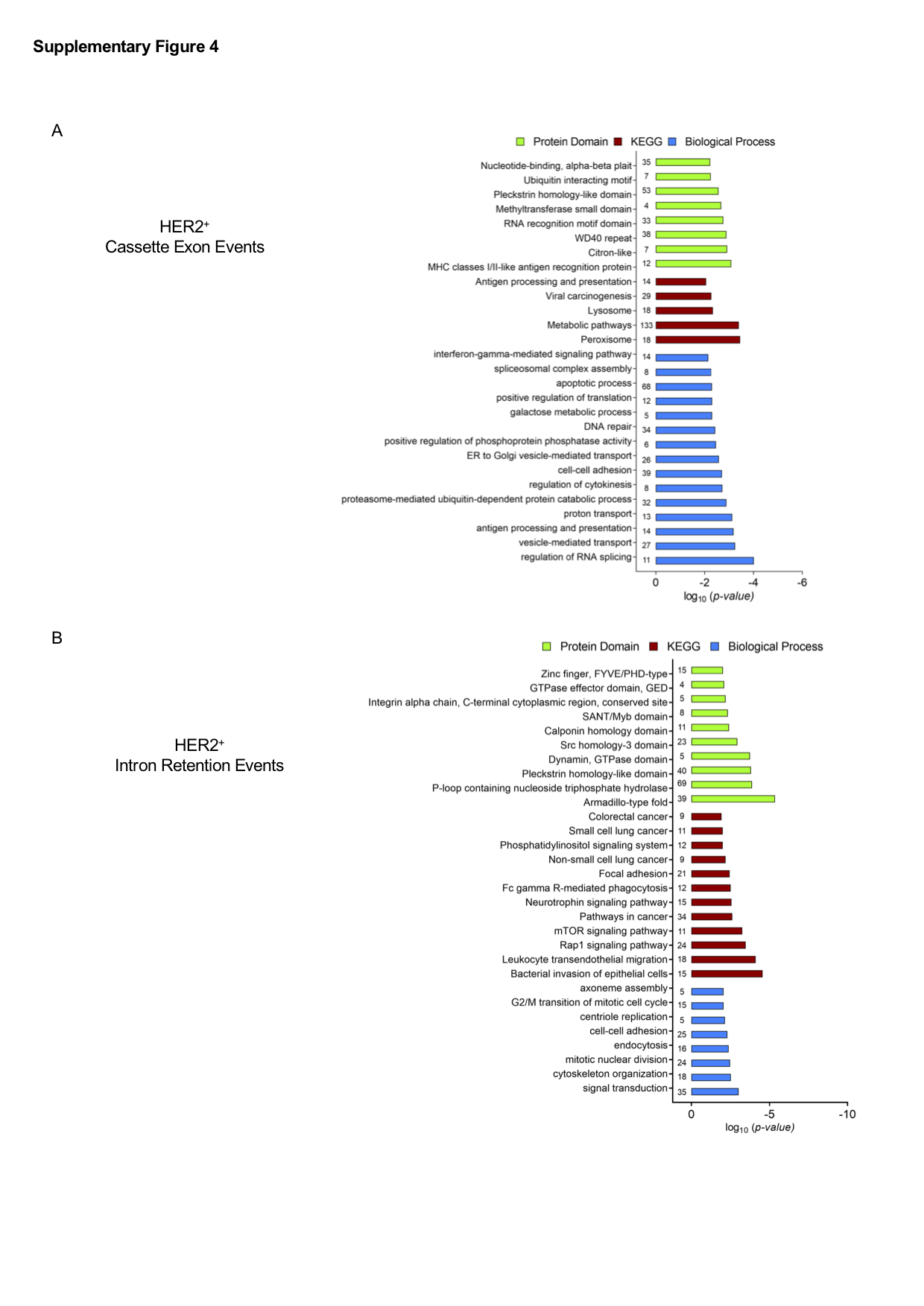

Supplement: Supplementary file 5 — Supplementary Figure 4 [file 41419_2021_3436_MOESM5_ESM.tif]

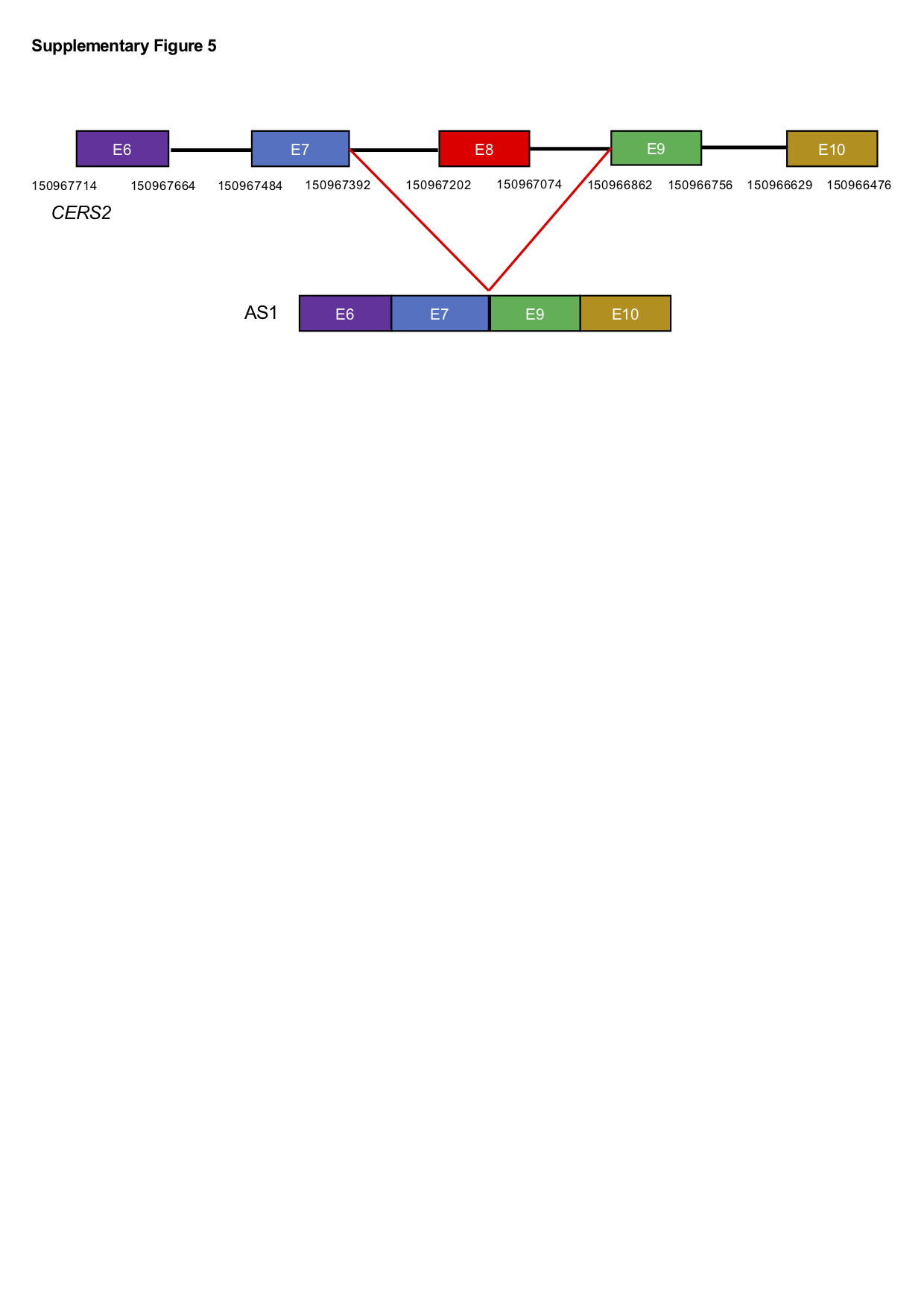

Supplement: Supplementary file 6 — Supplementary Figure 5 [file 41419_2021_3436_MOESM6_ESM.tif]

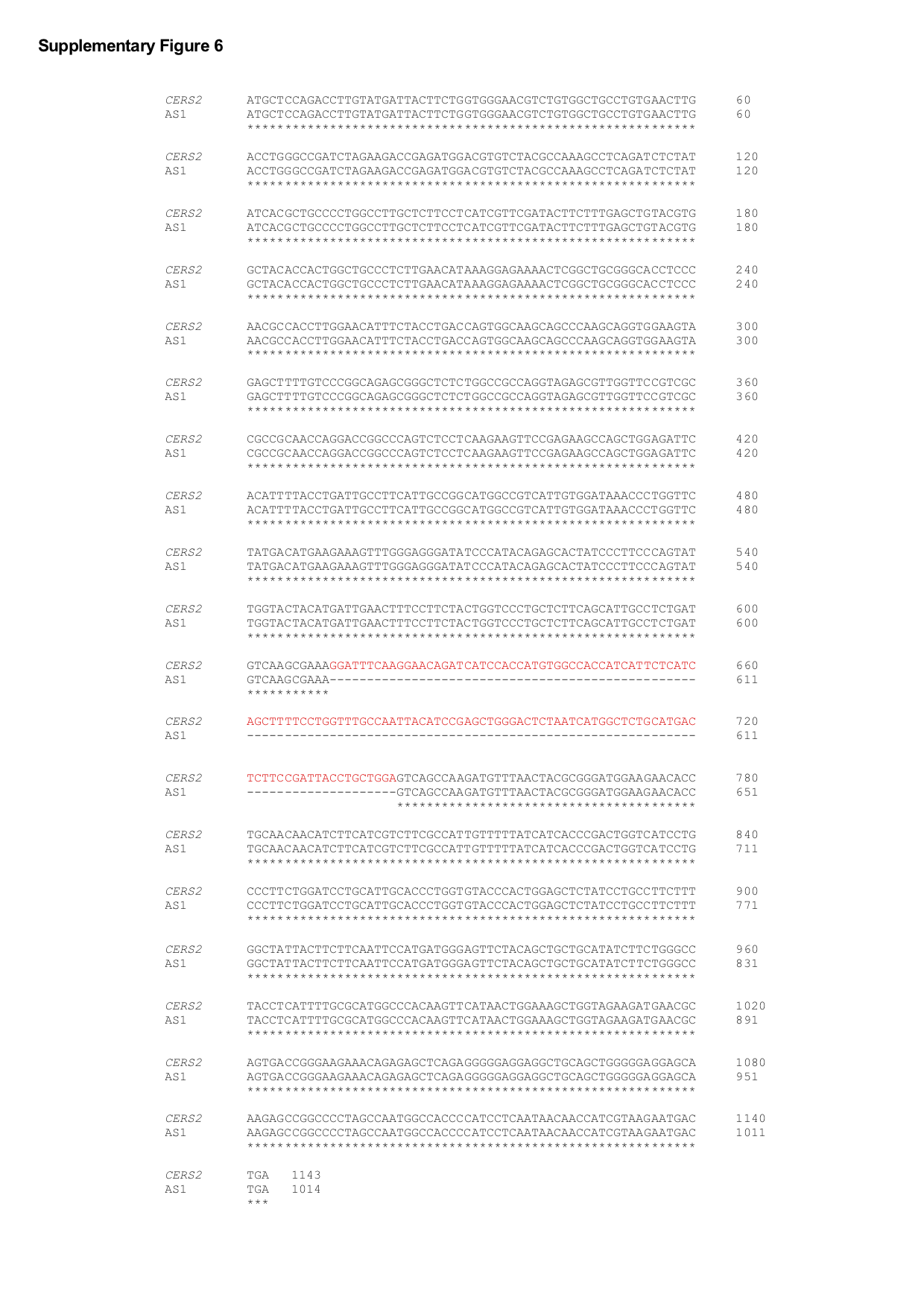

Supplement: Supplementary file 7 — Supplementary Figure 6 [file 41419_2021_3436_MOESM7_ESM.tif]

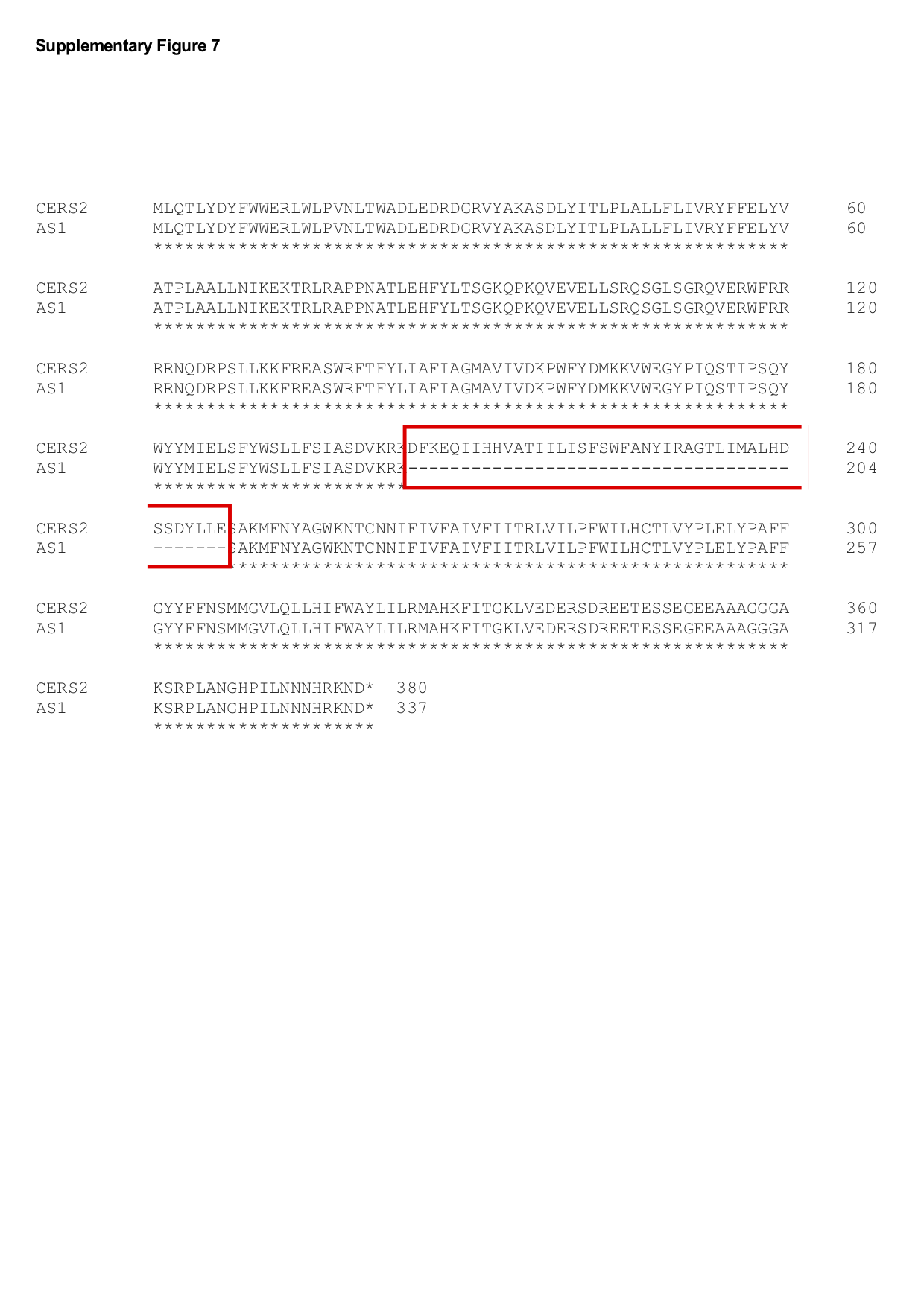

Supplement: Supplementary file 8 — Supplementary Figure 7 [file 41419_2021_3436_MOESM8_ESM.tif]

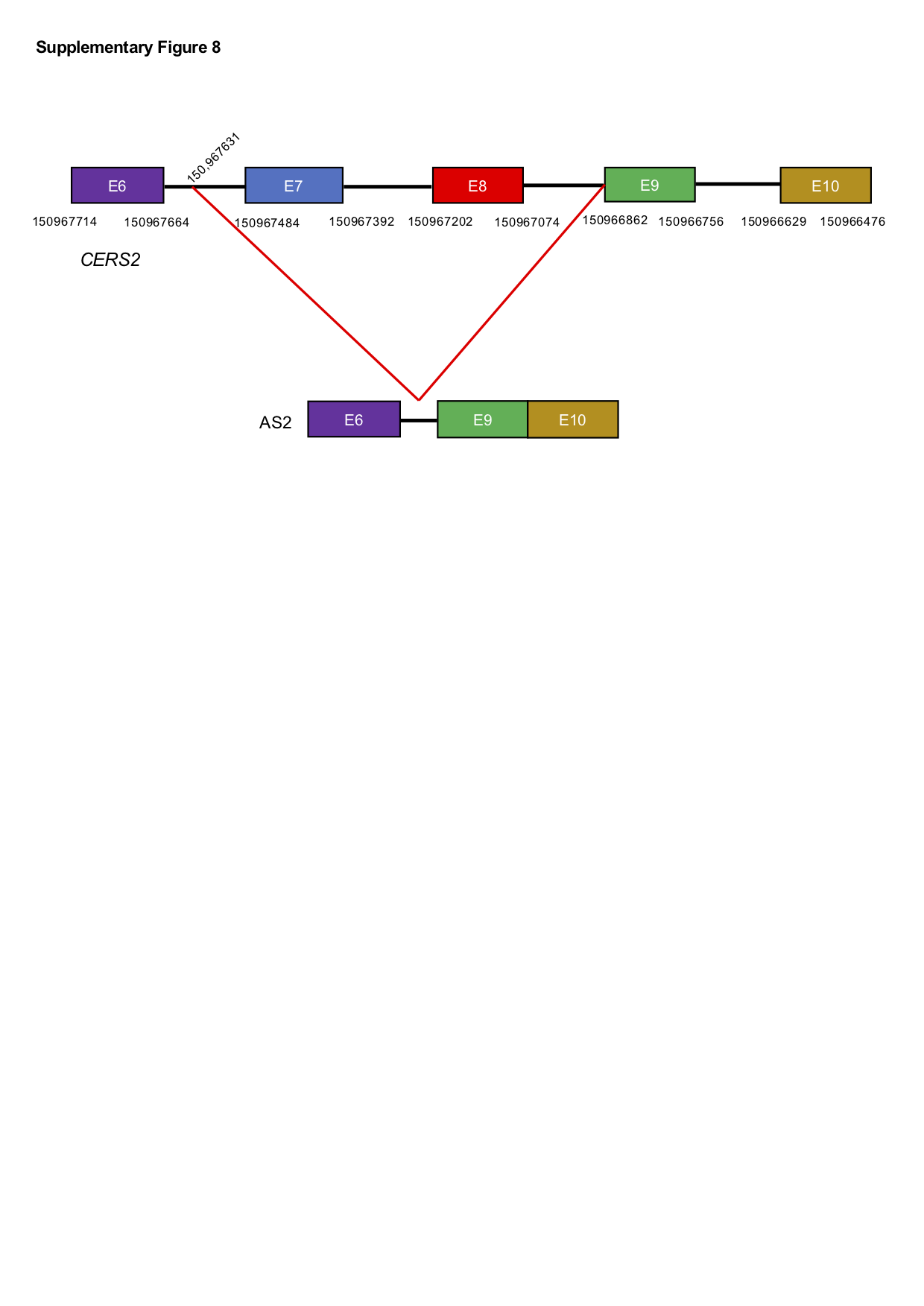

Supplement: Supplementary file 9 — Supplementary Figure 8 [file 41419_2021_3436_MOESM9_ESM.tif]

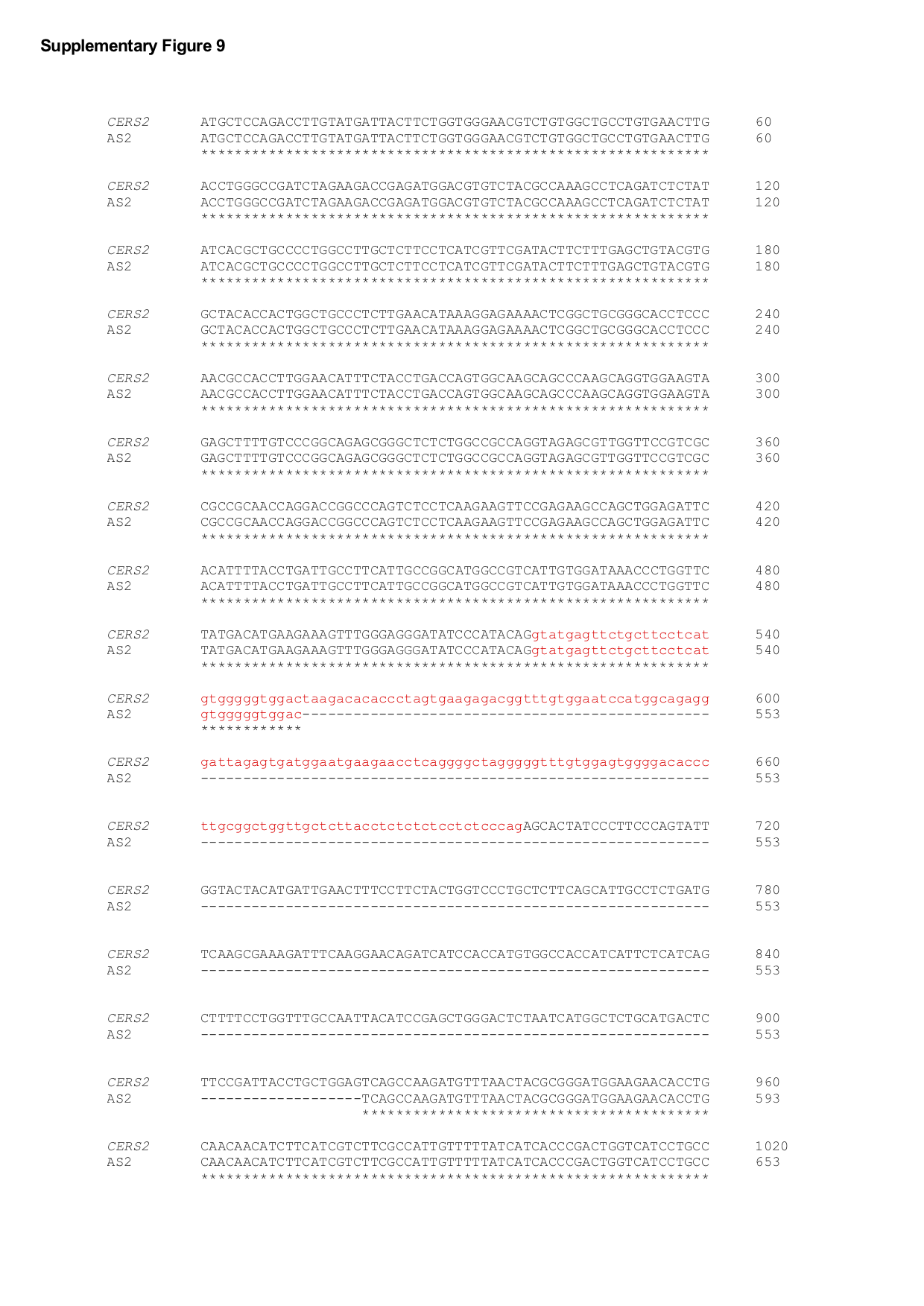

Supplement: Supplementary file 10 — Supplementary Figure 9 [file 41419_2021_3436_MOESM10_ESM.tif]

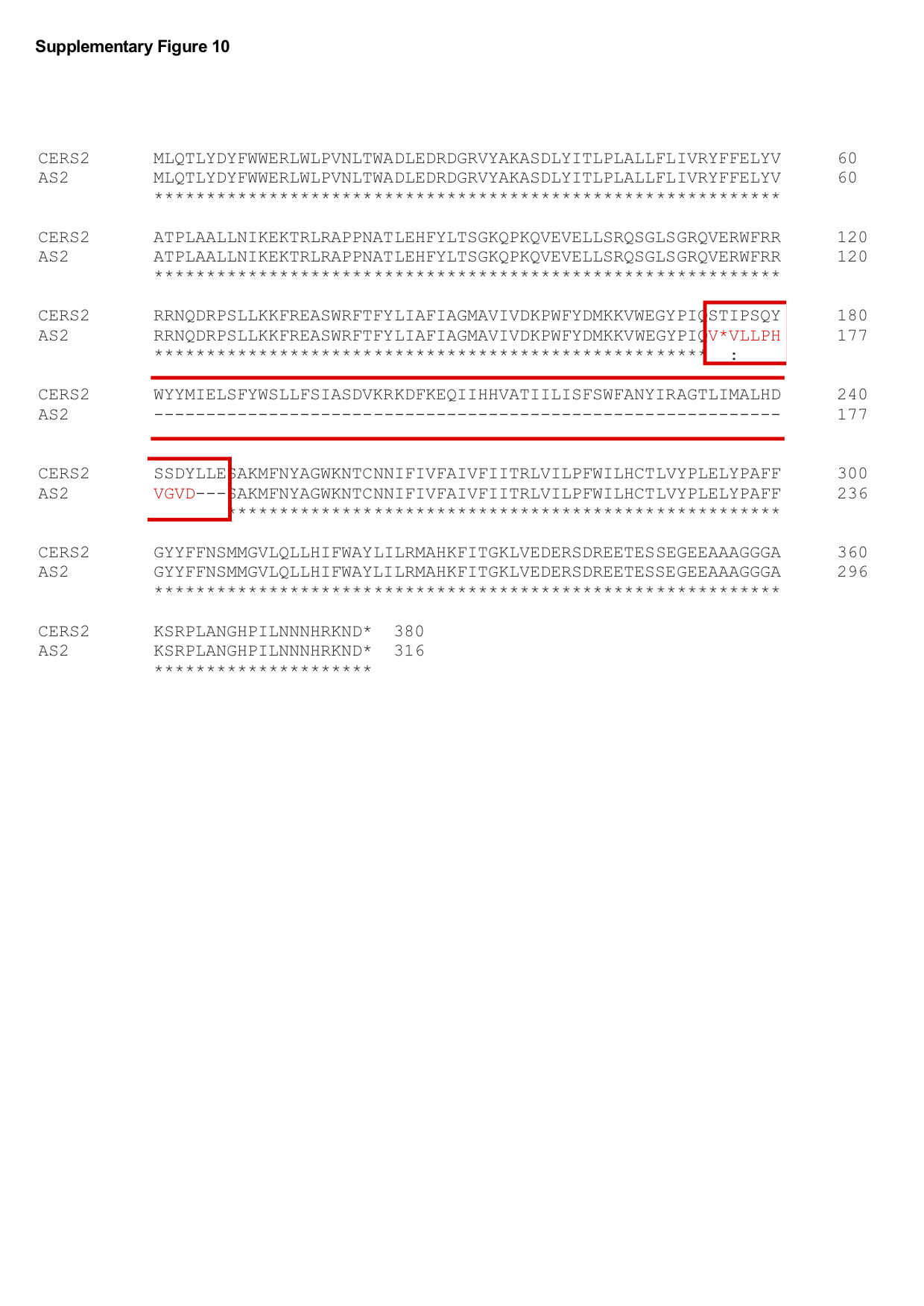

Supplement: Supplementary file 11 — Supplementary Figure 10 [file 41419_2021_3436_MOESM11_ESM.tif]

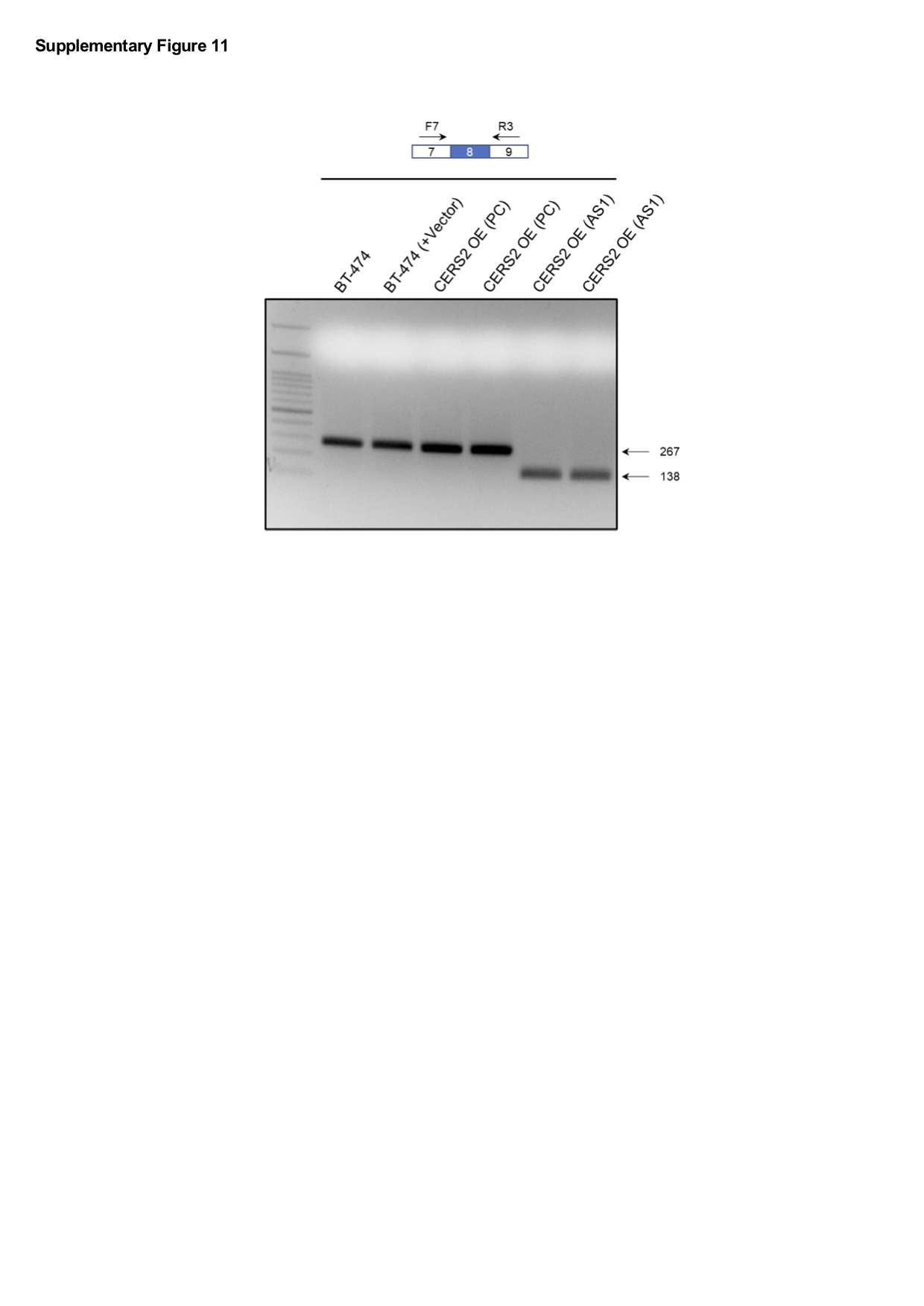

Supplement: Supplementary file 12 — Supplementary Figure 11 [file 41419_2021_3436_MOESM12_ESM.tif]

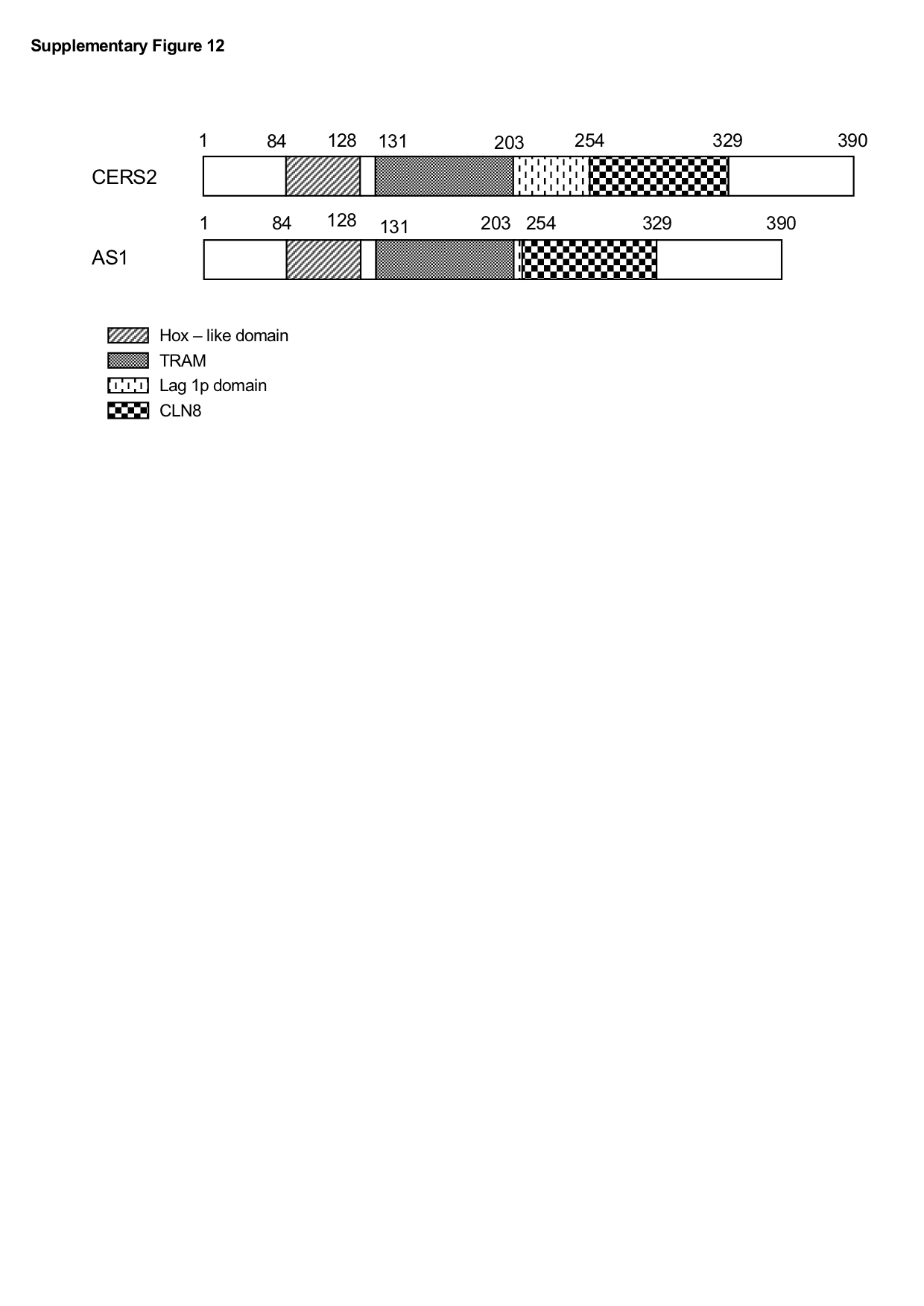

Supplement: Supplementary file 13 — Supplementary Figure 12 [file 41419_2021_3436_MOESM13_ESM.tif]

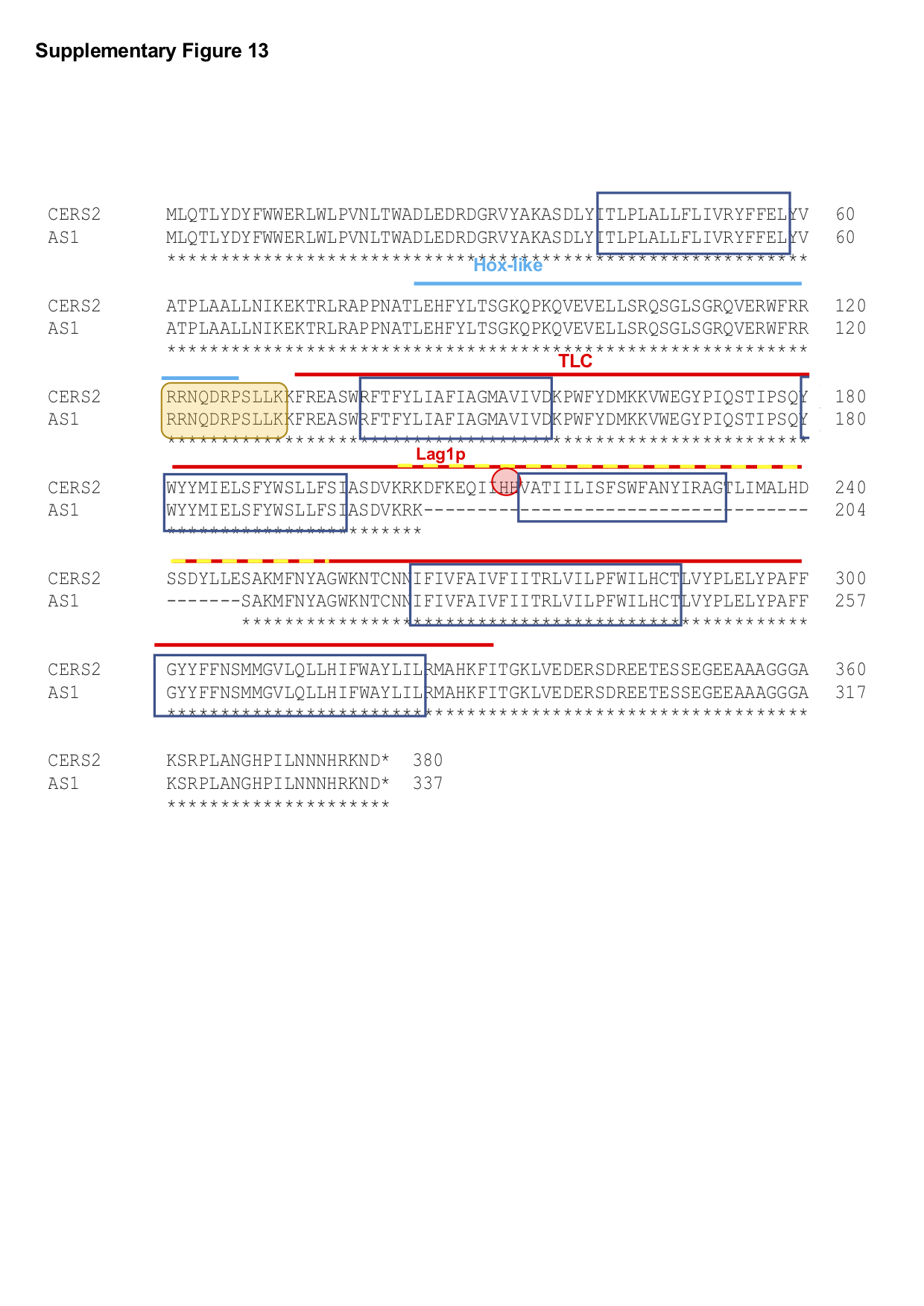

Supplement: Supplementary file 14 — Supplementary Figure 13 [file 41419_2021_3436_MOESM14_ESM.tif]

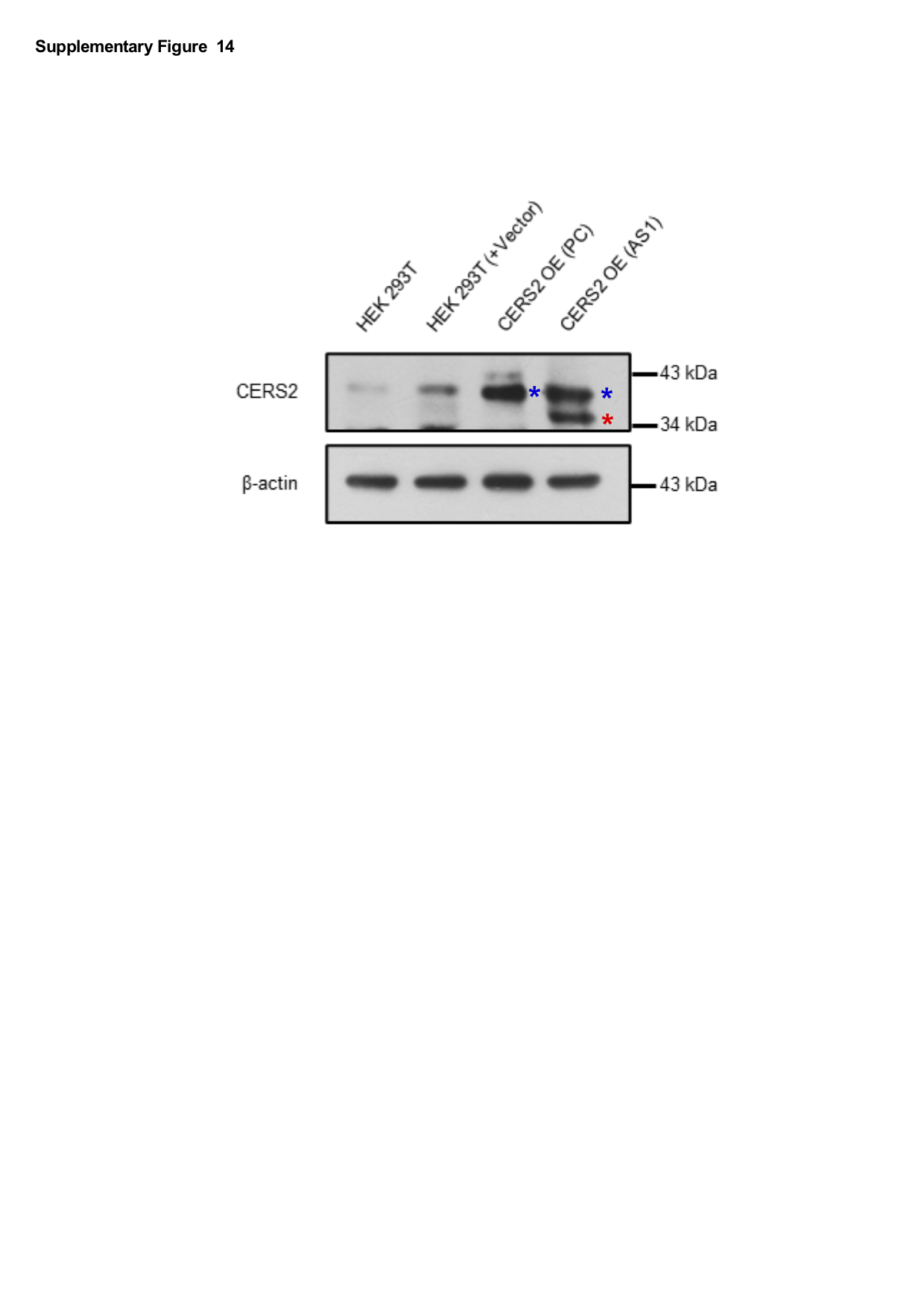

Supplement: Supplementary file 15 — Supplementary Figure 14 [file 41419_2021_3436_MOESM15_ESM.tif]

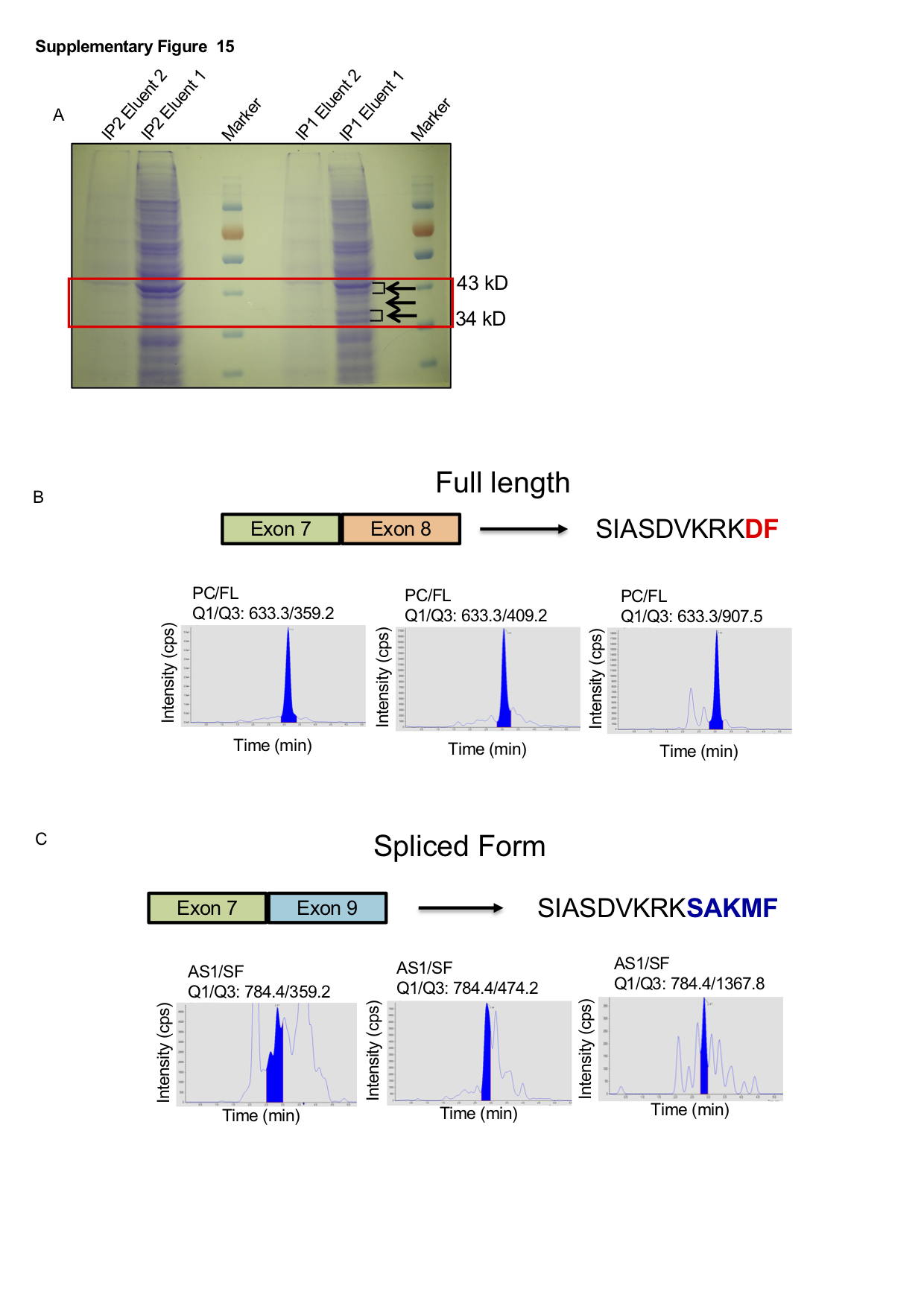

Supplement: Supplementary file 16 — Supplementary Figure 15 [file 41419_2021_3436_MOESM16_ESM.tif]

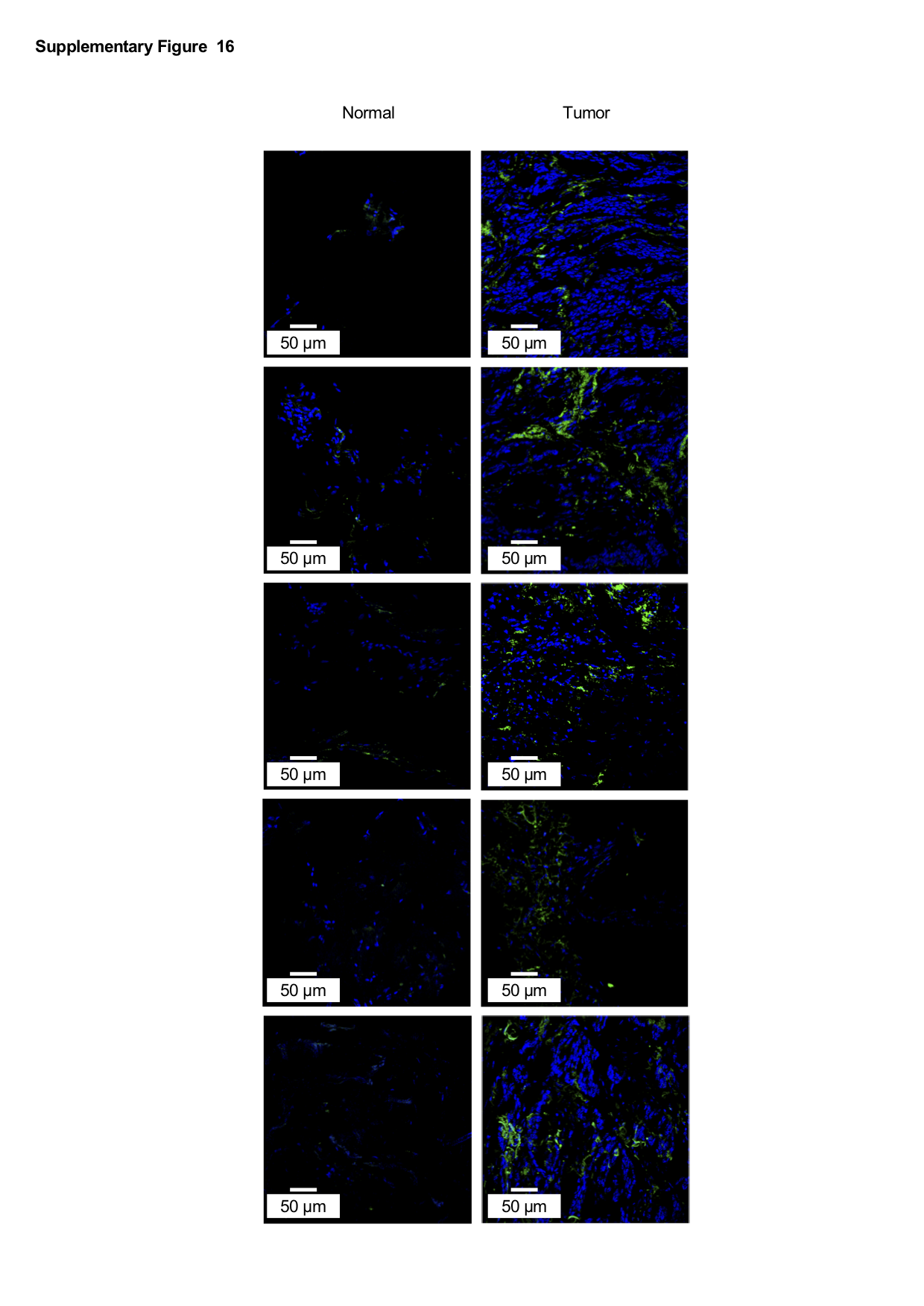

Supplement: Supplementary file 17 — Supplementary Figure 16 [file 41419_2021_3436_MOESM17_ESM.tif]

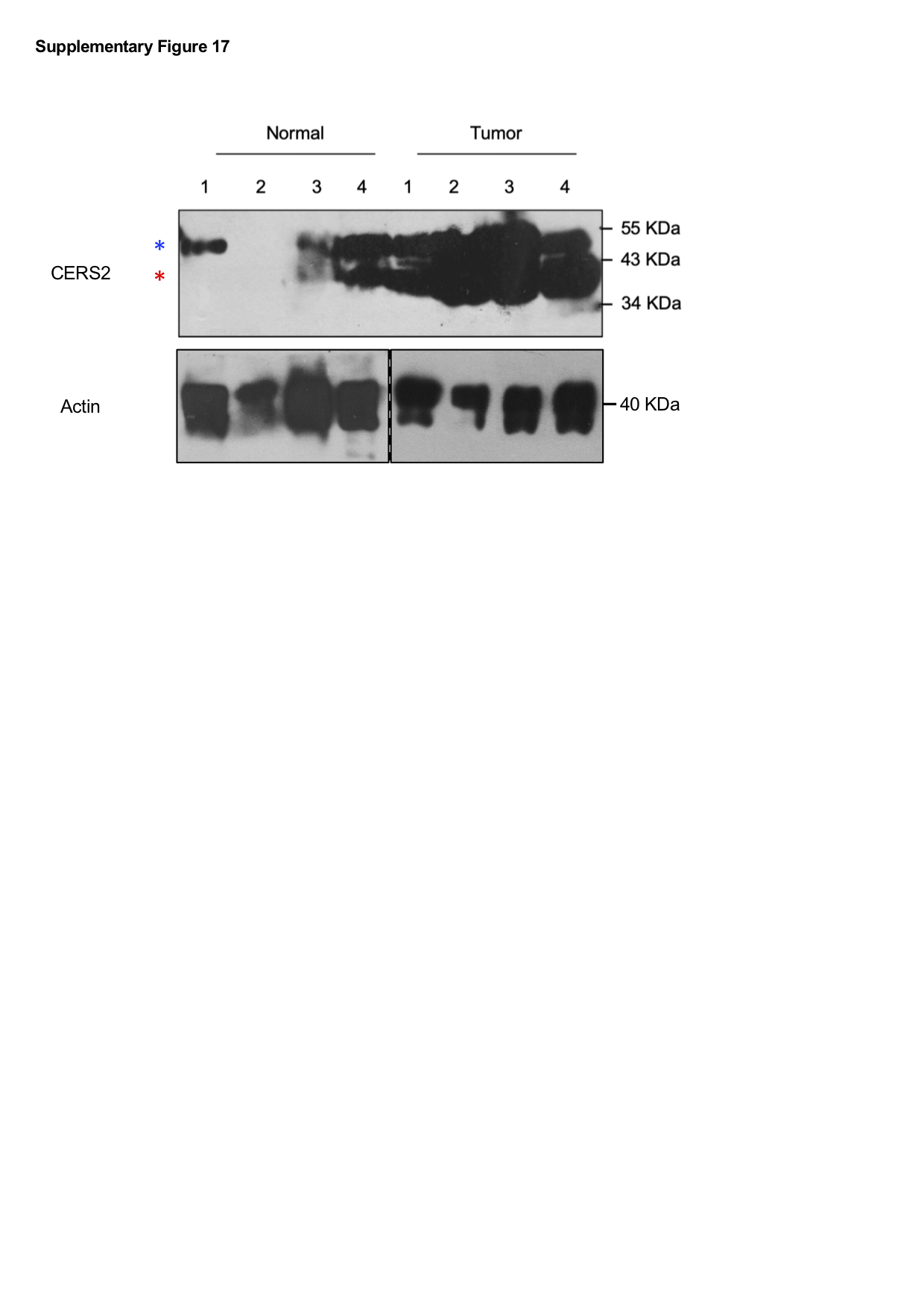

Supplement: Supplementary file 18 — Supplementary Figure 17 [file 41419_2021_3436_MOESM18_ESM.tif]

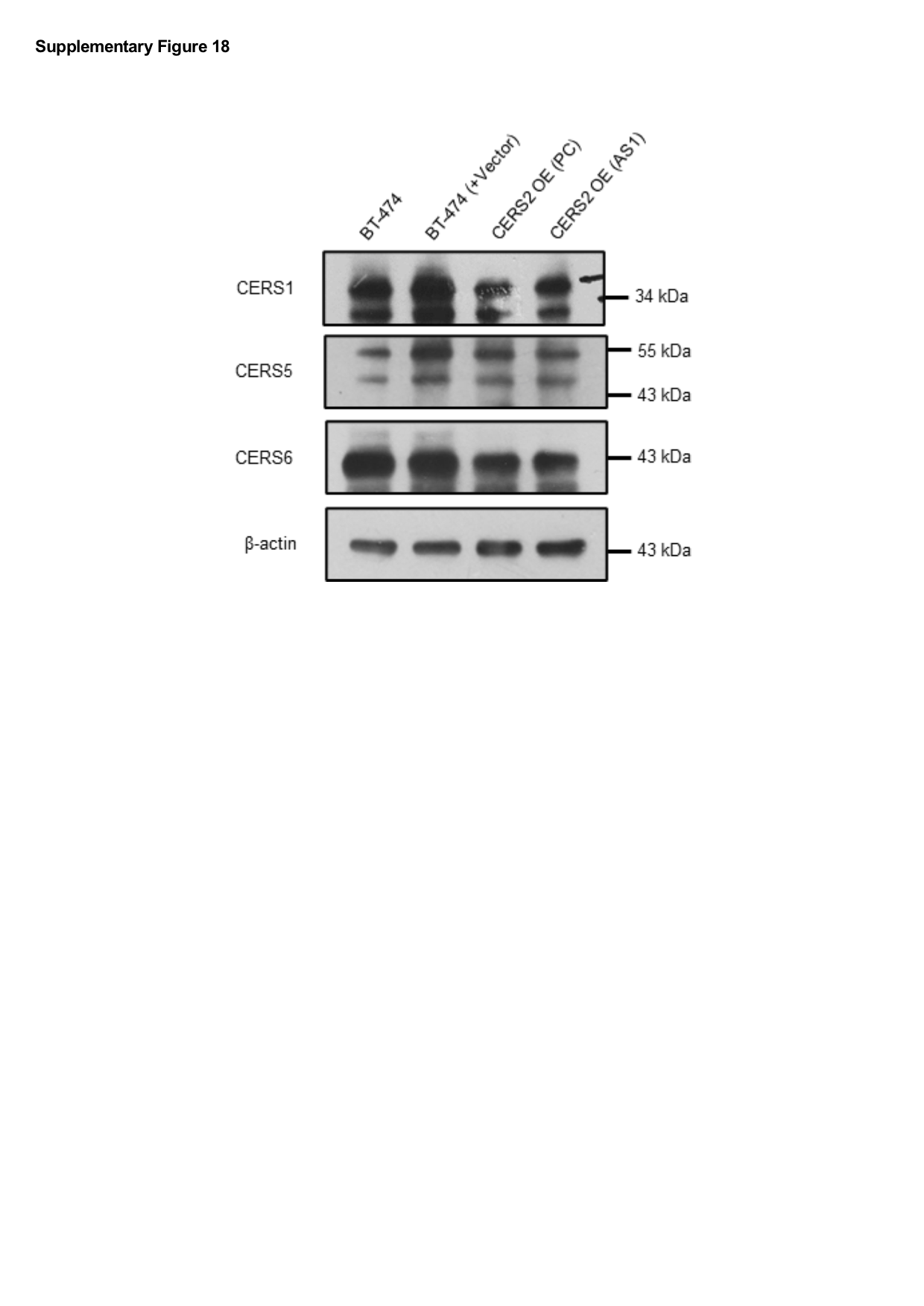

Supplement: Supplementary file 19 — Supplementary Figure 18 [file 41419_2021_3436_MOESM19_ESM.tif]

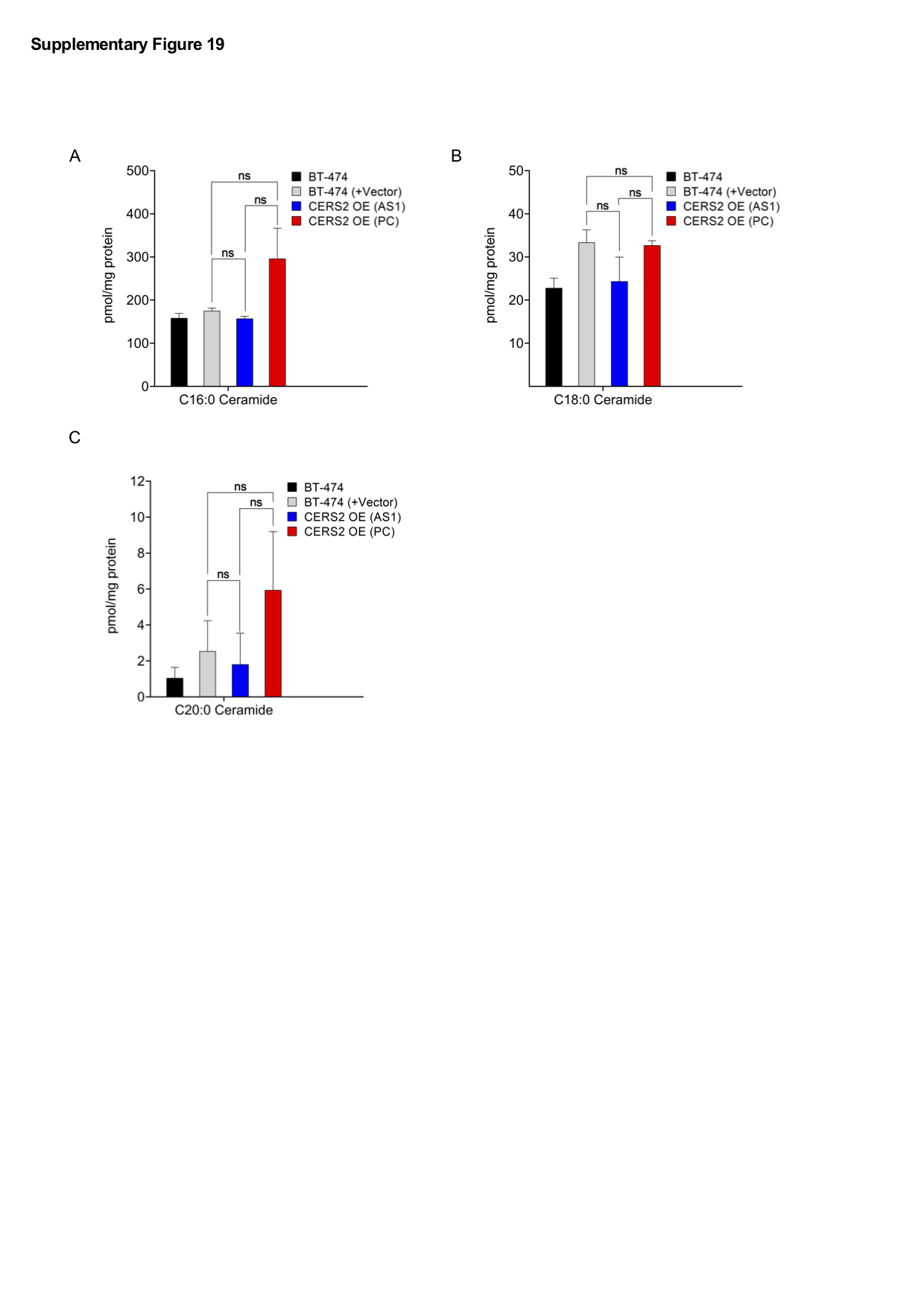

Supplement: Supplementary file 20 — Supplementary Figure 19 [file 41419_2021_3436_MOESM20_ESM.tif]

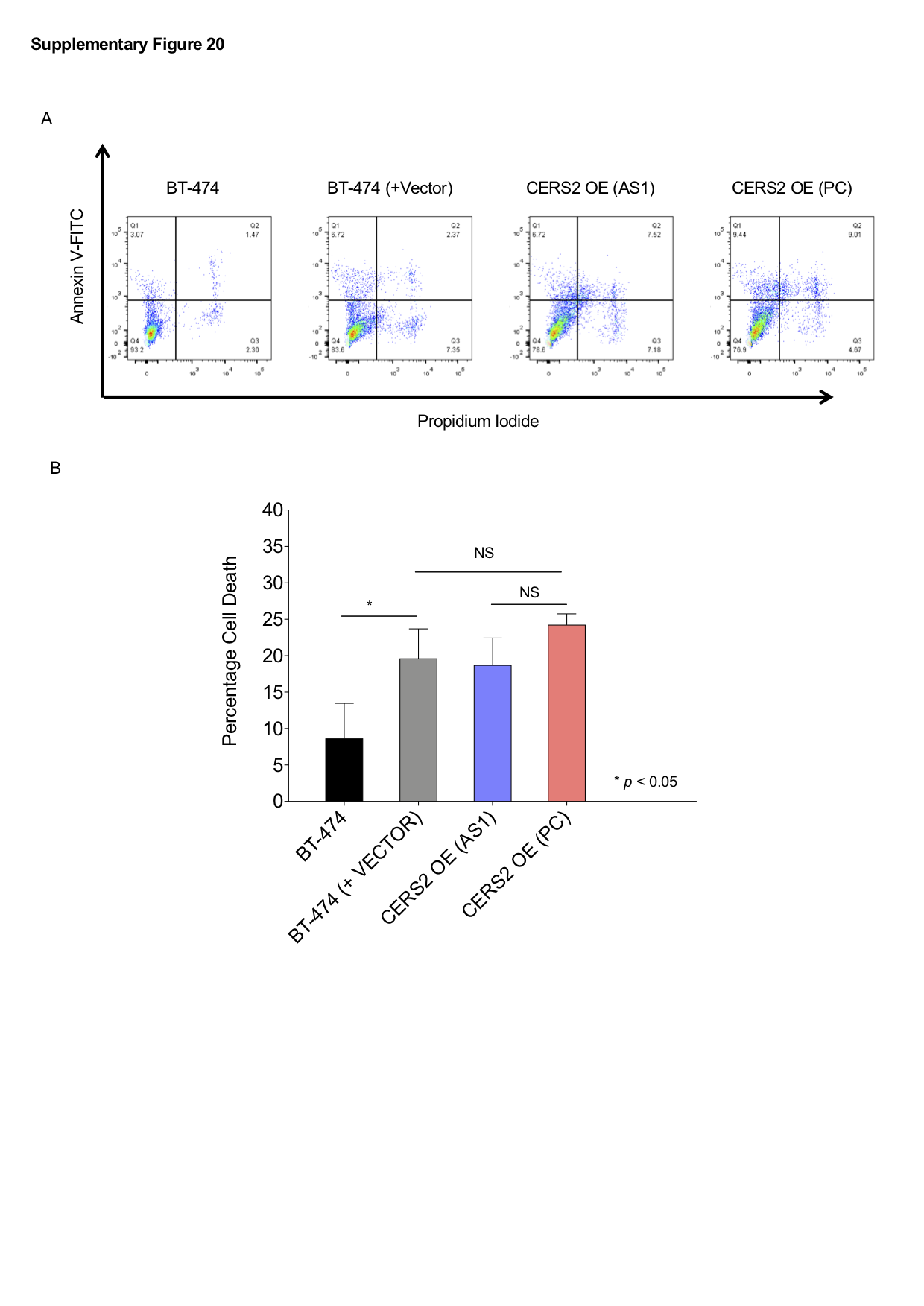

Supplement: Supplementary file 21 — Supplementary Figure 20 [file 41419_2021_3436_MOESM21_ESM.tif]
